# Hydrothermal synthesis of metal nanoparticles@hydrogels and statistical evaluation of reaction conditions’ effects on nanoparticle morphologies†

**DOI:** 10.1039/d4nr00581c

**Published:** 2024-09-06

**Authors:** Olivier Gazil, D. Alonso Cerrón-Infantes, Nick Virgilio, Miriam M. Unterlass

**Affiliations:** a Universität Konstanz, Department of Chemistry, Solid State Chemistry Universitaetsstrasse 10 78464 Konstanz Germany miriam.unterlass@uni-konstanz.de; b CREPEC, Department of Chemical Engineering, Polytechnique Montréal C.P. 6079 Succursale Centre-Ville Montréal Québec H3C 3A7 Canada; c CeMM-Research Center for Molecular Medicine of the Austrian Academy of Sciences Lazarettgasse 14 AKH BT25.3 1090 Vienna Austria

## Abstract

We report a facile green hydrothermal synthesis (HTS) of monoliths of hydrogels decorated with noble metal nanoparticles (NPs). The one-pot approach requires solely water, a polysaccharide able to form a hydrogel, and a salt precursor (M^*x*+^-containing) for the metal NPs. The polysaccharide fulfills three roles: (i) it acts as the reducing agent of M^*x*+^ to M^0^ under hydrothermal conditions, (ii) it stabilizes NPs surfaces, and (iii) it forms a hydrogel scaffold in which the metal NPs are embedded. The NPs’ localization in the hydrogel can be controlled through the gelation mechanism. Specifically, the NPs can either be located on and slightly under the surface of the hydrogel monoliths or in the volume. The former is found when a hydrogel monolith is crosslinked prior to HTS. The latter is observed when the HTS reaction mixture contains a polysaccharide dissolved in H_2_O, which forms a hydrogel upon cooling. Furthermore, we studied the influence of HTS conditions on NP shapes. To find significant levers towards morphological control, a set of HTS experiments featuring broad ranges of reaction conditions was performed. Subsequently, we employed statistical analyses with multivariate regression fits to evaluate synthesis parameter effects. Thereby, we can link the synthesis parameters of temperature, time, precursor concentration, heating rate, choice of metallic precursor, and type of biopolymer, to morphology descriptors such as diameter, circularity, and polydispersity index. The presented approach is *in fine* compatible with broad arrays of NPs and can in principle be modified for different chemistries, thereby providing a tool for quantitatively assessing morphological impacts of reaction parameters.

## Abstract 2

We report a hydrothermal one-pot approach using polysaccharides and salt precursors to synthesize and selectively localize metal nanoparticles in hydrogels, leveraging multivariate regression fits to further evaluate synthesis parameter effects.

## Graphical abstract

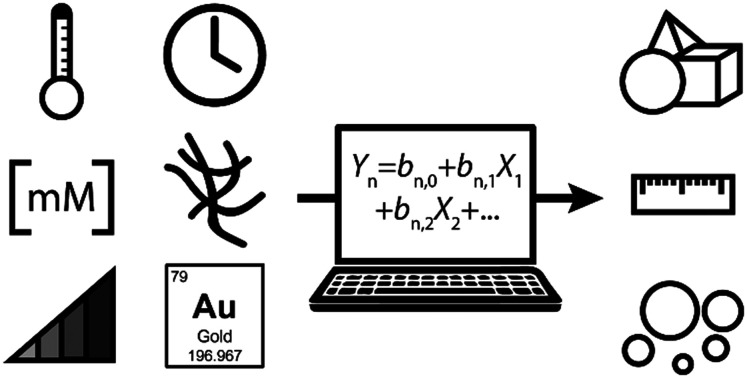

## Introduction

A nanoparticle (NP) is a roughly isometric object, *i.e.*, extending with similar length into all three directions in space, in the range of *ca.* 1–100 nm. Considering that atoms and chemical bonds lie in the size range of Ångstrøms (10^−10^ m), a particle in the range of 10^−9^–10^−7^ m (1–100 nm) will necessarily display a relatively high number of atoms at the surface (*N*_SA_). For instance, at an edge length of 10 nm, AgBr cubic nanoparticles feature *ca.* 25% (*N*_SA_/*N*_V_ ≈ 1/4) of their atoms at the surface.^1^ These high surface area-to-volume atom ratios (*N*_SA_/*N*_V_) apply to all NPs, irrespective of their chemical composition. Intriguing application-relevant physicochemical features arise, especially compared to the same bulk material. For instance, Pd^0^ NPs present much higher catalytic activities than a macroscopic piece of Pd^0^.^2^ This greater activity results primarily from a higher relative amount of surface atoms, as heterogenous catalysis requires adsorption of molecules at surface atoms.^3,4^ Furthermore and especially in lower nanorange particles, quantum size effects do manifest.^5^ In addition, the quantum confinement effect plays also a role for NPs composed of semiconductors.^6^ These effects generate intriguing optoelectronic phenomena, such as plasmon resonance.

No matter if an application of NPs is related to surface atoms or quantum effects, NP size and shape – comprehensively termed morphology – have to be well-controlled for a given application. Metal NPs for heterogeneous catalysis will for instance exhibit different activities and selectivities depending on NP shape, as different polyhedral shapes necessarily go hand in hand with a change in the number of faces and/or edges and/or vertices (and additionally for faces, a change in the shape of the face). An atom of a certain element within a given crystal structure, will exhibit a different number of dangling bonds and coordination geometries, depending if it is positioned on a face (and which face), an edge, or a vertex. Consequently, its propensity to adsorb small molecules and steric preference for adsorption will vary with position, which is encoded in the NP's morphology. Strong effects of NP morphology on optoelectronic properties have been observed: Schatz *et al.* have both experimentally shown and computationally studied the effect of NP anisotropy on the optoelectronic properties of Ag and AuNPs.^7^ Macroscopically, this, *e.g.*, strongly manifests in the colors of sols of Au^0^ NPs.^8^

In this study, we have set out to achieve three goals: first, we wanted to generate metal NPs within monolithic hydrogel matrices. Second, we aimed at developing maximally benign synthetic pathways towards these materials, notably focusing on using a minimum of additives, and by using water at increased temperatures as the reaction medium (*i.e.*, employing hydrothermal synthesis, HTS), in order to minimize both synthesis and processing complexity, and the number of steps. Third, we have set out to find levers for morphological manipulation and control that align with sustainable synthesis, *i.e.* resulting from reaction parameter variations alone (*e.g.*, temperature, time), without the need for chemical additives such as shape modifiers or control agents.

We have chosen monolithic materials consisting of metal NPs anchored in/on a support. Not immobilizing NPs but dispersing them in a fluid results in difficult recovery. Applications that benefit from supported NPs include heterogeneous catalysis, but also surface-enhanced Raman spectroscopy (SERS), which is capitalizing upon surface plasmon resonance of metal NPs. SERS can be done in a colloidal sol, but measurements are greatly simplified when metal NPs are anchored.^9^ (i) Rigid (inorganic) solids, (ii) flexible (polymer) solids, and (iii) gels, are the three major types of NP supports. Most used are rigid solids, such as metal oxides or zeolithes.^10,11^ Far less exploited are soft flexible solids such as polymers, elastomers or foams, *e.g.*, polydimethylsiloxane, crosslinked polystyrene-type polymer networks, or polyurethane.^12–14^ Soft supports provide the ability to absorb/soak up fluids containing a substrate that one wishes to catalytically convert into a product. NP-decorated flexible foams in particular feature the possibility to absorb the reactants and expel the products (*i.e.*, soak and squish) over thousands of cycles, which can *e.g.*, as we have shown recently, be exploited for automation using robotic arms.^15^ NPs@flexible sponges, *e.g.*, NPs@polyurethane or NPs@cellulose, were used in a batch-type process for cross-couplings,^16,17^ reductions,^18,19^ and hydrogenations.^17^ For flow settings, the potential of sponges has been both mentionned^16,17^ and demonstrated.^18^ Third, gels can be employed as supports, combining the transport characteristics of liquids with solid-like features, *e.g.*, monolithic shapes. This unique nature is a consequence of gels being co-continuous structures of a fully percolated liquid phase within a fully percolated solid. Typically, NPs@hydrogels are synthesized by incorporating NPs in a gel-former solution, followed by gelation^20^ (note that NPs can also act as cross-linkers for polymers, as was reported for AuNPs cross-linking thiolated hyaluronic acid).^21^ Alternatively, a metal precursor can be loaded into the gel and reduced *in situ*.^20^

The synthesis of supported metal NPs comprises (i) the synthesis of the metal NPs, (ii) the synthesis of the support, and (iii) the anchoring of the NPs in/on the support. In terms of eco-friendliness, all three aspects have to be considered.^22^ Regarding the metal NPs synthesis, these are most commonly synthesized *via* wet routes involving the reduction of a metallic precursor (*e.g.* HAu^III^Cl_4_ for Au^0^NPs) with often harmful reducing agents such as NaBH_4_, N_2_H_4_, or DMF.^5^ Consequently, it is desired to replace the classical reductants by eco-friendlier ones. It has been shown that saccharides, due to their carbonyl moieties, are able to reduce metallic ions to metal NPs.^23–25^ Polymeric derivatives of saccharides, polysaccharides, which are often of natural origin and are considered non-toxic eco-friendly materials, have been reported as part of green reducing systems, *i.e.*, in combination with a second reducing agent. Examples include glucose with starch,^23^ NaBH_4_ with cellulose^26^ and chitosan,^27^ or ascorbic acid with gum arabic.^28^ A second reducing agent is often needed since polysaccharides alone are often too weak of a reducing agent to generate M^0^ from M^*x*+^. Interestingly, hydrothermal synthesis (HTS, *i.e.*, employing liquid water at *T* > 100 °C as medium) confers stronger reductive properties to polysaccharides and can help improve the efficiency and reproducibility of NP synthesis.^29^ Several reports of metal NP HTS use natural polysaccharides as reducing agents, *e.g.*, alginate,^25,30^ chitosan,^25^ gum Arabic,^31^ and starch.^32^ All of these works exclusively focus on Ag^0^ NPs using AgNO_3_ as precursor. First, expanding the approach to other metals, which we tackle in this contribution, seems necessary. Second, polysaccharide reductants have been exclusively used in solution. We are not aware of any examples of HTS of metal NPs@hydrogels. One-pot syntheses of various NP@graphene hydrogels have been reported, however, these require additional reducing agents (*e.g.* hydroxyproline^33^ or triethylenetetramine^34^). Here, we intend to directly synthesize the NPs reduced by and within a three-dimensional polysaccharide network support (*i.e.*, in hydrogels) for directly generating supported NPs. By performing both the NP synthesis and their immobilization in a green fashion in water as medium, the materials’ eco-friendliness is strongly enhanced. Note that we recently reported the HTS of metal NPs anchored on polyurethane sponges as reduction catalysts.^15^ Commercial polyurethane (PU) acts itself as a reduction catalyst under HT conditions, without need for any additional reducing agent. However, PU foams are industrially fabricated from petroleum-derived monomers and using not necessarily “green” methodologies. In contrast, polysaccharides as used here are extracted from renewable resources.

Metal NPs@hydrogels are of interest for biomedical applications, *e.g.*, by combining the ability of hydrogels to mimic the hydrated environment (*i.e.* extracellular matrix) of cells,^35^ with the anti-microbial activity of AgNPs, or the electrical conductivity of AuNPs.^25,35^ Furthermore, hydrogels provide transport characteristics which, when combined with the physicochemical features of the embedded metal NPs, can be applied beyond biomedical materials. For instance, in combination with AuNPs for surface-enhanced Raman spectroscopy (SERS), selective diffusivity of molecules smaller than the mesh size of the hydrogel network has been shown to limit pollution and thus maximise the SERS signal of the desired molecules.^36^ Precise control over the localization of NPs in hydrogels, possible *via* an *in situ* interfacial synthesis of NPs in the hydrogels (*e.g.* AuNPs),^37^ displayed increased catalytic activities in the reduction of 4-nitrophenol to 4-aminophenol.

Different morphologies of otherwise chemically identical metal NPs go hand in hand with different combinations and relative sizes of crystal facets, which in turn strongly affect the physicochemical properties of NPs. The morphologies of metal NPs depend chiefly on the crystal structure of the metal but can be influenced through synthesis. For instance, additives, through adsorption unto certain facets, can be used to inhibit their growth (hence these facets persist and increase relatively to non-blocked facets). Furthermore, the growth rates of certain facets can be influenced through physical parameters such as temperature.^38^ In this work, we hypothesized, during HTS of metal NP@hydrogel that (i) the polysaccharides can also act as morphology-modifying additives, that (ii) the viscoelastic properties and permeability of the hydrogels can alter the diffusion of metal ions compared to synthesis in liquid medium, hence influencing the NP growth, and that (iii) the elevated temperatures in HTS can have an impact on the obtained morphologies by influencing growth rates and potentially generating growth instabilities/inhomogeneities. Herein, we investigate the effects of synthesis parameters on NPs morphologies and thereby provide a first proxy for the morphological variety of metal NPs generated by HTS. For maximal rationality of such an interpretation and in order to be able to estimate the significance of this interpretation, we have employed statistical tools. We believe that the statistical evaluation and workflow applied herein is of use for the nanoscience community, when studying nanoparticle morphology evolution.

Design of experiments (DoE) to prepare nanomaterials is efficient at identifying the important synthesis variables, gives insight into the NPs growth mechanism, and helps optimize and control the synthesis process.^39^ However, this has yet to make its way to many NPs studies. On one hand, the nanomaterial scientists who try to understand mechanisms and trends behind the synthesis of NPs evaluate the effect of one variable at a time (OVAT method), which greatly limits the reach of their investigations.^40^ One can think of the numerous studies about the Turkevich method exploring the full range of parameters for this synthesis route.^41,42^ On the other hand, the applied scientists, such as the ones in nanomedicine, who want to optimize the use of nanomaterials in their domain, tend to use DoE rather as an optimization tool to control a specific characteristic of NPs (such as size,^43^ band gap energy,^44^ UV-vis absorbance, or drug-loading).^39^ A typical example of DoE on NPs morphologies was realized by Kumar *et al.* on AuNPs, where they found that the Au/citrate ratio is an important parameter controlling the size and size distribution of AuNPs.^45^ A study performed by Burrows *et al.* digs deeper into the silver-assisted growth of Au nanorods and gives insights into the role of silver and ascorbic acid on the aspect ratio of these nanomaterials.^46^ While eight experimental variables were used in a fractional factorial design, many challenges still remain in the understanding of this kind of seeded-growth approach in general.^46^ Machine learning is also a powerful tool to design new nanomaterials and would help with the collection of large datasets.^40,47^ Investigations focusing on nanomaterials synthesis, either one variable at a time or by DoE, tend to focus on a single parameter, and we did not find any studies dealing with multiple changes in morphologies as we do in this study.^39,40^

## Results and discussion

**Scheme 1:**
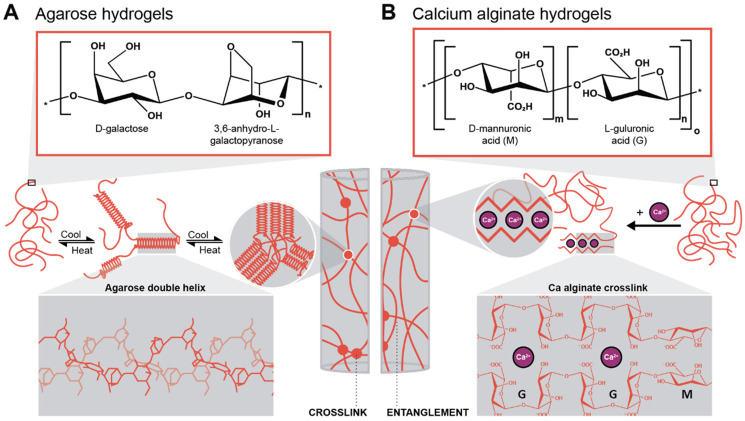
Gelation mechanisms of the two hydrogels investigated in this work. (A) Chemical structure of the agarose repeating unit and gelation mechanism of the polymer with double-helix conformation (3D structure highlighted in the inset), adapted from ref. 49. (B) Chemical structure of the two alginic acid repeating units (multiblock copolymer) and the gelation mechanism with Ca-alginate, *i.e.*, by cross-linking through divalent cations (inset: egg-box structure of Ca-alginate), adapted from ref. 51.

To generate supported metal nanoparticles (NPs), we aimed at using polysaccharide hydrogels as both reducing agent and support under HT conditions. We have employed two polysaccharides with different gelation mechanisms. The first system is based on an aqueous solution of a polysaccharide in which a metallic precursor is solubilized. Note that the precursor mixture is a gel at rt and a solution when heated above the gelling temperature. The gelled mixture is heated up to the HT regime, NPs form, and upon cooling, the polysaccharide forms a hydrogel (thermal gelling). We herein used agarose as a polysaccharide that forms hydrogels thermally. Agarose contains alternating α-(1→3)-linked d-galactose and β-(1→4) 3,6-anhydro-l-galactopyranose units and gels by a combination of chain entanglement and helix-coil transitions of agarose chains (Scheme 1A).^48,49^ Specifically, by heating agarose in H_2_O above approx. 80 °C, the agarose chains form random coils. Upon cooling, when *T* ⪅ 80 °C is reached, segments of neighboring chains associate to form helix conformations, and neighboring helix segments pack subsequently into small aggregates acting as crosslinks. Additionally, entanglements exist between segments of the chains that have not assumed helix conformations, also contributing to the gel state. In summary, under HT conditions (*T* > 100 °C), the agarose chains are solubilized in superheated H_2_O, and the metal NPs formed by reduction of metal salts under HT conditions are formed inside an agarose solution and not inside a gel. The NPs-containing hydrogel then forms upon cooling of this NPs dispersion. Consequently, NPs within agarose gels are expected to be evenly distributed throughout the entire volume of the gel. The second approach was to plunge an already crosslinked hydrogel, herein alginic acid crosslinked with Ca^2+^ ions forming Ca-alginate, into a solution containing the metallic precursor. Alginic acid is a linear copolymer containing (1→4)-linked β-d-mannuronate (M; a carboxylic acid featuring derivative of mannose) and α-l-guluronate (G; a carboxylic acid featuring derivative of gulose) groups (Scheme 1B). This polymer displays blocks of G-residues, blocks of M-residues, or alternating M- and G-residues. The ratio between M- and G-residues plus block configuration depends largely on the brown algae species, from which alginic acid has been extracted, and will *in fine* dictate the materials properties.^50^ Alginate gels are forming so-called egg-box structures, where Ca^2+^ ions are chelated by O-donor atoms of the M and G rings, *i.e.*, by both ether oxygen atoms and CO_2_^−^ groups (Scheme 1B).^51^ In contrast to the agarose system, which is gelling upon cooling, the Ca-alginate system is already a gel prior to HTS and does not dissolve under HT conditions, since the polymer chains are strongly crosslinked with Ca^2+^ (one would need to displace the Ca^2+^ to reverse crosslinking, such as by chelating the divalent ions with sodium citrate for example). Consequently, the Ca-alginate system is expected to feature a higher number of NPs near the surface of the hydrogel specimen, *i.e.*, near the interface of the cylindrical specimen with the surrounding precursor solution, as the metal precursors have to diffuse into the gel.

As a synthetic starting point, we employed the conditions that we have recently reported for the HT reduction of metal salts by polyurethane (PU) foams and anchoring of the formed metal NPs on the PU foams.^15^ Precisely, we employed aqueous solutions containing the respective metal precursors (0.25 mM HAuCl_4_, or AgNO_3_, or Na_2_PdCl_4_). In the case of calcium alginate hydrogels, preformed cylinders of *V* = 1 mL and dimensions *r* = 0.5 cm and *h* = 1.3 cm were prepared (see ESI for preparation and Fig. S1† for aspect). The cylinders were plunged into 4 mL of the respective aqueous precursor solution. In the case of agarose, agarose powder was dissolved at a temperature above the gelation temperature and the aqueous metal precursor solution was added to form an overall volume of 5 mL of solution. Once the solution was homogeneous, it was quickly cooled down. Both systems were allowed to react at *T*_R_ = 120 °C for a reaction time *t*_R_ = 3 h in a steel autoclave (see ESI† for details).

**Fig. 1:**
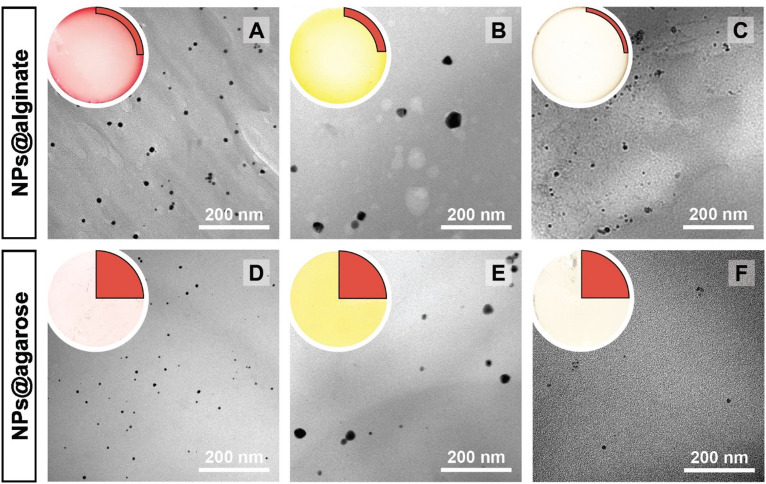
Macroscopic aspects of NPs@hydrogels and NP morphologies. Top left circle of each panel: photograph of the cross section of the respective specimen, as cut with a razor blade with the highlighted region showing where sampling for TEM was performed in a 90° sector. Angular section of each panel: corresponding TEM image of the NPs@hydrogel. All NPs@hydrogels were synthesized at *T*_R_ = 120 °C, *t*_R_ = 3 h and [M^*x*+^] = 0.25 mM. NPs were synthesized in calcium alginate (A–C) and agarose hydrogels (D–F), respectively. The types of metal NPs are: Au^0^ (A&D), Ag^0^ (B&E) and Pd^0^ (C&F). Higher magnifications of specimens A–C are presented in Fig. S2.†

**Fig. 2:**
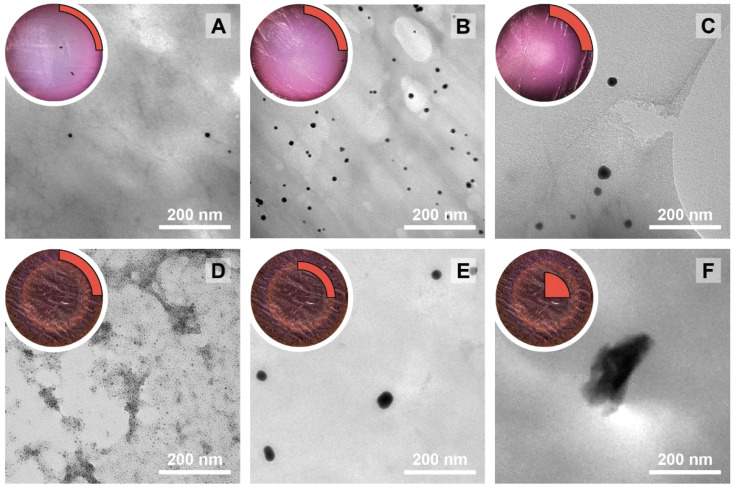
Study of the gold precursor concentration over the nanoparticles’ morphologies in Ca-alginate hydrogels. Optical micrographs (reflected light) and nanoparticles morphologies of the AuNPs@alginate. The synthesis conditions are *T*_R_ = 120 °C, *t*_R_ = 3 h and [Au^3+^] = 0.083 (A), 0.25 (B), 0.75 (C) and 2.5 mM (D–F). Note the three distinct zones at 2.5 mM AuNPs@Ca-alginate, from which samples were taken for TEM investigations: outside zone (D), in-between zone (E) and inner zone (F).

The NPs@Ca-alginate materials (Fig. 1A–C) feature an intensely colored ring at the rims of the cross sections and a gradient of strongly colored to weakly colored from rim to center. The color is strongly indicative of successful NP formation and the observed colors fit well with the plasmon resonances/typical coloring of NPs of the respective metals (Au: red-violet; Ag: yellow; Pd: gray-black). The strongly colored rings at the rims indicate that the majority of NPs has indeed formed near the surface of the Ca-alginate gel. The gradient towards weaker coloring in the direction of the center indicates that the metal salts diffused into the specimen, but that the majority is reduced close to the interface with the surrounding solution. Accounting for this fact, TEM sampling was done on the rim (highlighted in red in Fig. 1). To compare the different NP@alginate materials, we determined average NPs diameters *D* and the circularity *circ* from TEM images, analyzing >100 NPs per material (see ESI† for details). *D* is defined as a segment that would pass through the middle of an equivalent area circle, while *circ* is a measure of how round and smooth a shape is (1: perfect circular shape; 0: highly non-circular shape) in 2D digital image analysis (eqn (S1)†). Note that from conventional 2D electron microscopy images, one can only extract circularity (2D) but not of sphericity (3D). From TEM image analysis, it becomes clear that AgNPs tend to be larger and less circular (*D*_Ag_ = 31 ± 9 nm; *circ*_Ag_ = 0.93) than Au- and PdNPs, which form both smaller and highly circular NPs (*D*_Au_ = 12 ± 2 nm, *circ*_Au_ = 0.97; *D*_Pd_ = 11 ± 2 nm, *circ*_Pd_ = 0.98). For NP@agarose (Fig. 1D–F), the results are strikingly different: the cross-sections of the gels display complete color homogeneity. This goes in line with our initial hypothesis that the metal ions are reduced in agarose solutions in the HT regime, and that the resulting NP sols have gelled upon cooling to rt. The NPs are significantly smaller than those obtained in calcium alginate hydrogels: *D*_Au_ = 9 ± 1 nm, *D*_Ag_ = 13 ± 6 nm, and *D*_Pd_ = 4 ± 1 nm, but are similarly circular: *circ*_Au_ = 0.95, *circ*_Ag_ = 0.93, and *circ*_Pd_ = 0.99. To gain further insight into the diffusion of the precursor metal ions into the preformed Ca-alginate hydrogels under the employed mild HT conditions, we have synthesized a series of AuNPs@alginate materials of increasing precursor concentration (Fig. 2): [HAuCl_4_] = 0.083, 0.25, 0.75, and 2.5 mM. Interestingly for the highest concentrations, 3 distinct concentric circles (or concentric cylinders in 3D) are observed in the cross-sections (highlighted in yellow in Fig. 2D–F): an orange outer annulus, a purple intermediate annulus, and a reddish middle circle (see ESI Fig. S3† for magnified views and sharper colors). The circle and annuli we observe remind of periodic precipitations known as the Liesegang phenomenon. Liesegang rings form when two precursors, with one being a gel phase, react and form a colloidal precipitate with a reacting front.^52^ These structures are governed by a delicate equilibrium between transport phenomena of the reducing agent (or the metallic precursor) into the gel, and the reaction kinetics of NPs formation. The mechanistic origins for Liesegang ring formation – diffusion of reactants in a gel and NP formation kinetics – are also at work in our case and it is thus not surprising to observe these ring patterns for NPs@alginate (Fig. 2). Optical microscopy at high magnification (Fig. S3†) provides further strong evidence of selective localization within the hydrogel. The distinct zones of nanoparticle growth – outer annulus (orange), in-between annulus (purple), and inner core (reddish-pink) – correspond to particles with varying diameters. This phenomenon arises from a delicate balance between precursor reduction at the hydrogel interface and precursor diffusion. By controlling reaction kinetics, one may impart particle localizations, akin to the Liesegang phenomenon. Opposed to the slow heating rate that leads to concentric zones of Au^0^ by allowing HAuCl_4_ to migrate into the hydrogel without immediate reduction, is Exp 21, presented later. In this experiment, PdNPs are exclusively localized at the monolith's surface. To further analyze the NPs’ localization, cross-sections of samples were immobilized in epoxy resin and the surface was microtomed for scanning electron microscopy (SEM) analysis (Fig. S4†). Due to the small size of NPs and the necessity for them to be precisely on the surface of a microtome cut to be visible, drawing absolute conclusions about selective localization using SEM is difficult.

In a study on AuNPs@poly(vinyl alcohol) hydrogels, Yoon *et al.* observed Liesegang rings, albeit at the micron-scale, and additionally noted two important aspects: (i) larger particles were found farther away from the hydrogel's surface, and (ii) spherical particles were observed near to the surface, while faceted (*i.e.*, more anisotropic) ones were seen towards the core of the hydrogels.^53^ Similar to these observations, for HTS performed at *c* = 2.5 mM, the average NP size gradually increases from the outside of the hydrogel (Fig. 2D) towards the inside (Fig. 2F) with diameters of 4 ± 1, 29 ± 5 and 240 ± 20 nm for specimen shown in Fig. 2D, E and F, respectively. At the same time, we observe particles with increasing anisotropy as we move away from the surface and towards the center of the hydrogel (*cf.*Fig. 2D*vs.*2E*vs.*2F). The increasingly less spherical morphologies towards the center of the AuNPs@Ca-alginate specimen, in line with observed spatial localization of increasingly non-spherical morphologies by Yang and Pan,^30^ inspired us to look for synthesis parameters that would support non-spherical morphologies in the HTS of metal NPs@hydrogels.

To uncover these parameters and their effects, we decided to employ a design of experiments (DoE) approach. DoE is a structured approach that aims to find the relationships between input and output variables by first outlining a systematic testing protocol based on statistical approaches. The data is then collected and analyzed to provide information about the studied system in an efficient way. Consequently, DoE is, compared to parameter studies that investigate a high number of parameter combinations, a faster and less experiment-intensive method for exploring the effects of experimental parameters on a certain output variable.

In the DoE study employed herein, the factors (*X*_*n*_) that we decided to study were synthesis temperature and time, precursor concentration, heating rate, the choice of metallic precursor, and the type of hydrogel (see Table 1). In our case, they are categorical variables with either qualitative or quantitative levels, which are the different possible synthesis conditions. These parameters were chosen for the following reasons: we expected both the nucleation and growth mechanisms to be impacted by synthesis temperature and time (*X*_1_ and *X*_2_). For the concentration (*X*_3_), we expected that it would impact the degree of supersaturation^54^ and might affect the number of initially formed nuclei,^55^ hence affecting the NPs diameter. Concerning the heating rate (*X*_4_), we were expecting its change to affect the number of initially formed nuclei, but also to affect anisotropy for potentially favoring/suppressing out-of-equilibrium shapes for the smaller nuclei having higher surface energy.^56^ For example, in the classical Turkevich reduction towards AuNPs, Ding *et al.* have shown that an increase in heating rate causes an accelerated nucleation, which leads to a reduction in size.^57^ Note that in order to enable both faster and controlled heating rates, we switched from batch autoclaves to a microwave reactor. Finally, the choice of precursor (*X*_5_) and hydrogel (*X*_6_) were expected to impact the synthetic outcome, since the different chemistries involved must display different signatures such as different kinetics. As output parameters, we decided to focus on the diameter *D* (*Y*_1_), the circularity *circ* (*Y*_2_ – *cf*. ESI, eqn (S1)†) and the polydispersity index *PDI* (*Y*_3_ – *cf*. ESI, eqn (S2)†). All three are important morphological descriptors. We deemed the synthetic outcomes as desired: (i) small average particle sizes (small *D*), (ii) rather monodisperse NP distributions (small *PDI*, *Y*_3_) and high anisotropy (low *circ*, *Y*_2_). Note that all three output parameters were extracted from TEM images, measuring >100 NPs in all cases, and up to >300 NPs in several cases. Sampling was performed such that realistic averages of the specimen were obtained, *i.e.*, by taking samples from several regions in a monolith (see ESI† for details). Note furthermore that TEM was selected for extracting the morphology descriptors *Y*_1_, *Y*_2_, and *Y*_3_ over other techniques (such as UV-Vis spectroscopy, for instance, that probes larger quantities of NPs), as information beyond the average NP diameter, such as anisometry or output parameters minimal and maximal values, would be less straightforward to extract than from TEM images. Another technique that includes high numbers of NPs, namely powder X-ray diffraction (PXRD), was employed for the initial set of samples synthesized at 120 °C. Most PXRD curves did only give amorphous halos arising from the hydrogel, but no sharp reflections. Only samples synthesized with [HAuCl_4_] ≥ 0.75 mM display the characteristic reflections of crystalline fcc Au^0^ (see Fig. S5†). Importantly, the authors note that there are no dependencies between output parameters extracted from the study, which is a requirement for a DoE study design. An anisotropic particle (*i.e.*, low *circ*) can theoretically be obtained at any size and circular particles with a specific diameter can be either monodisperse or polydisperse – these responses should be independent.

**Table 1:** Experimental parameters used in the DoE phase

Symbol	Predictors and responses	Level	Unit
*X*_1_	Synthesis temperature	100 (1), 120 (2), 150 (3), 180 (4)	°C
*X*_2_	Synthesis time	1 (1), 5 (2), 30 (3), 180 (4)	Min
*X*_3_	Precursor concentration	0.083 (1), 0.25 (2), 0.75 (3), 2.5 (4)	mM
*X*_4_	Heating rate	As fast as possible (AFAP; 1), 20 (2), 5 (3)	°C min^−1^
*X*_5_	Metal precursor	HAuCl_4_ (1), AgNO_3_ (2), Na_2_PdCl_4_ (3)	—
*X*_6_	Hydrogel choice	Alginate (1), agarose (2)	—
*Y*_1_	Diameter	—	nm
*Y*_2_	Circularity	—	—
*Y*_3_	Polydispersity	—	—

Experiments were designed using the DoE software Azurad®, employing a screening study (aka fractional factorial DoE) in order to identify the major contributors to the selected responses (*Y*_1_, *Y*_2_, and *Y*_3_). This specific type of DoE allows the user to perform a reduced number of experiments by *screening* out the statistically irrelevant ones and is therefore usually used in the initial stages of experimentation. The software suggested the following 17 experiments to be carried out (Table 2). After carrying out the optimized experiments chosen by the software, a non negligible fraction led to non-exploitable results (rows featuring ‘*N*’ in the last column in Table 2, *i.e.*, experiments 2, 10, 11, 13, 14, 15, and 17). The non-exploitability consisted of, for example, significant agglomeration of the particles or destruction of the polysaccharide supports. The most interesting example of non-exploitable yet interesting result within the intended DoE is shown in Fig. S6.† For instance, under conditions (*X*_1_ = 180 °C, *X*_2_ = 1 min, *X*_3_ = 0.75 mM, *X*_4_ = 5 °C min^−1^, *X*_5_ = HAuCl_4_, *X*_6_ = agarose) the agarose was degraded, *i.e.*, undergoing hydrothermal carbonization, to carbon dots (Fig. S6B†). Intriguingly, at otherwise identical reaction conditions, carbon dots only formed in the presence of metallic NPs (seen for both gold and silver NPs), while the same conditions without particles led to a whitish suspension that cannot form a gel anymore (Fig. S6C†). In line with the latter example, some of the experiments suggested by the software gave intriguing results highlighting the marked effects that the variation of ‘simple’ synthesis parameters (*e.g.*, *T*, *t*) can have. Yet, for these results, certain response parameters are impossible to quantify, which is necessary for a DoE evaluation. For instance, if no NPs are formed, one could assign a diameter such as 0 nm, or if the NPs strongly agglomerate, one could just give the diameter of the agglomerate, but these arbitrary data points would lead to adulterated conclusions. Consequently, the fractional factorial DoE computed by Azurad® could not be completed. In principle, this could have been avoided by better exploring the experimental space and making sure that all the conditions could be applied successfully. However, this would have been limiting in our goal of finding peculiar conditions that would impart unexpected morphologies to the particles – one could have stayed for example at a temperature around 120 °C and *t*_R_s ≤ 30 min. However, this would have impeded the discovery of interesting particle morphologies at *e.g.* 180 °C. Consequently, a more conservative redesign of the DoE would have been compatible with the core principle of this technique, yet at the cost of not discovering unusual effects of the reaction parameters on the NPs’ morphologies.

**Table 2:** Factors and success rates for the DoE screening experiments

Experiment	*X*_1_	*X*_2_	*X*_3_	*X*_4_	*X*_5_	*X*_6_	Success (Y/N)
1	2	2	2	1	2	2	Y
2	4	4	4	1	2	2	N
3	3	4	1	2	1	2	Y
4	1	1	2	2	3	2	Y
5	1	2	3	2	2	1	Y
6	2	3	4	2	1	1	Y
7	1	3	1	3	2	2	Y
8	2	4	1	3	3	1	Y
9	3	1	2	3	2	1	Y
10	4	1	3	3	1	2	N
11	2	1	3	2	1	2	N
12	3	1	4	2	2	1	Y
13	4	2	1	2	3	1	N
14	1	3	1	2	2	2	N
15	4	3	2	1	1	1	N
16	1	4	3	1	2	1	Y
17	1	1	4	1	3	2	N

Instead of discarding the generated data, we decided to exploit the results using a statistical analysis of significant synthesis parameters impacting NPs morphology. To this end, an additional set of 15 random experiments (by generating randomized levels for each parameter) were performed, taking into account the previously gained knowledge of the failed experiments (*i.e.* limiting the synthesis time to a shorter span, 1 and 5 min, for the higher temperatures, 150 °C and 180 °C). The 8 initial conditions were also added as experiments in the study (excluding the 2.5 mM HAuCl_4_). In the end, exploitable results were obtained for a set of 31 experiments (ESI, Table S1†).

**Fig. 3:**
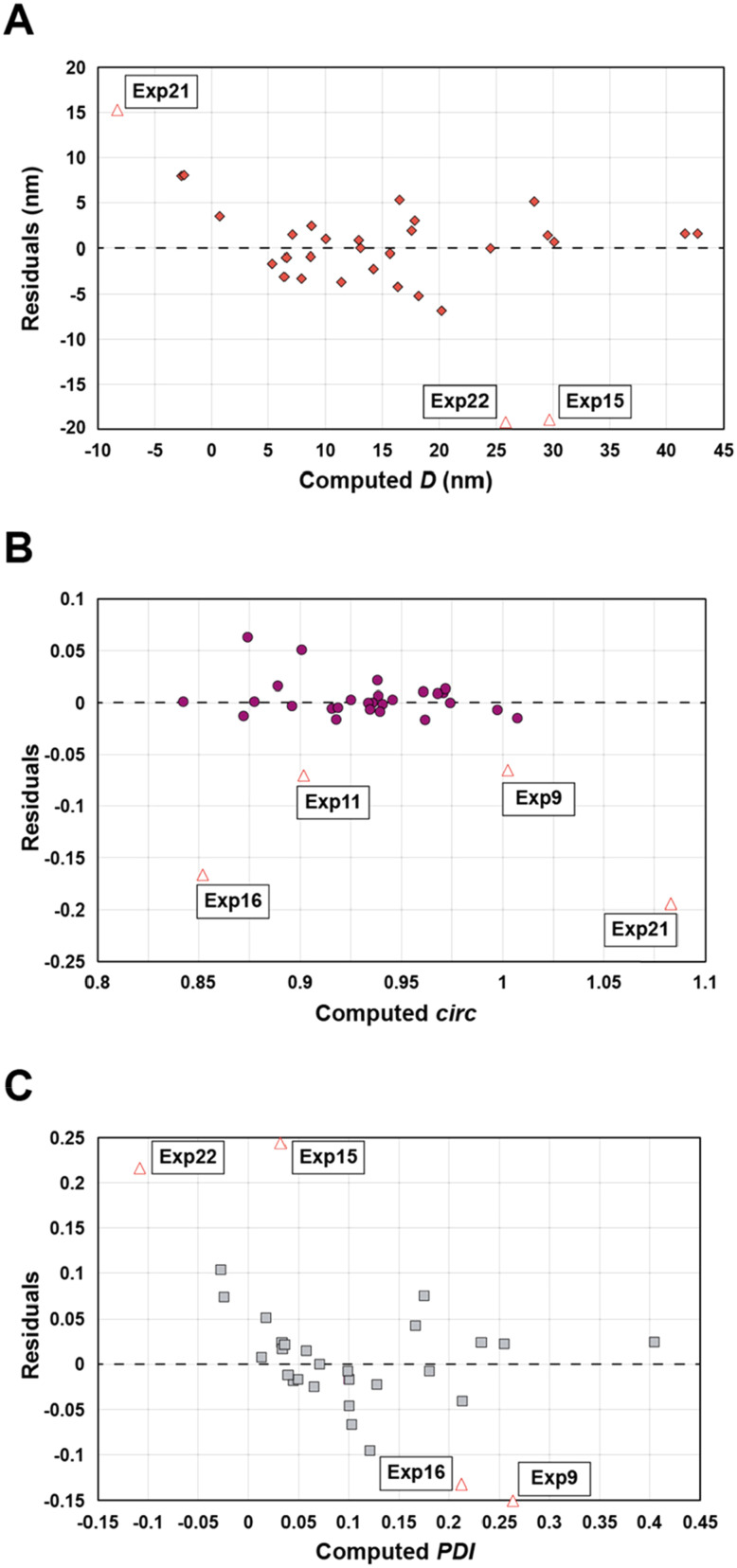
Residuals graphs for *D* (A), *circ* (B) and *PDI* (C) with the experiments 9, 11, 15, 16, 21, and 22 identified and highlighted as red triangles in each graph they are considered outliers.

Statistical analyses were performed on this data set by fitting multivariate regressions (see ESI for precisions alongside eqn (S3)†). We assumed the factors’ effects to be additive. Before settling with a certain regression, refining the model was necessary and required the identification and removal of outliers. Therefore, residuals analysis was performed, and data points that deviated significantly from the overall pattern were flagged. Experiments (Exp – different from the DoE experiments) 15, 21, and 22 were considered outliers for *D*, Exp 9, 11, 16, and 21 were considered outliers for *circ*, and Exp 9, 15, 16, and 22 for the *PDI* (see Fig. 3).

**Fig. 4:**
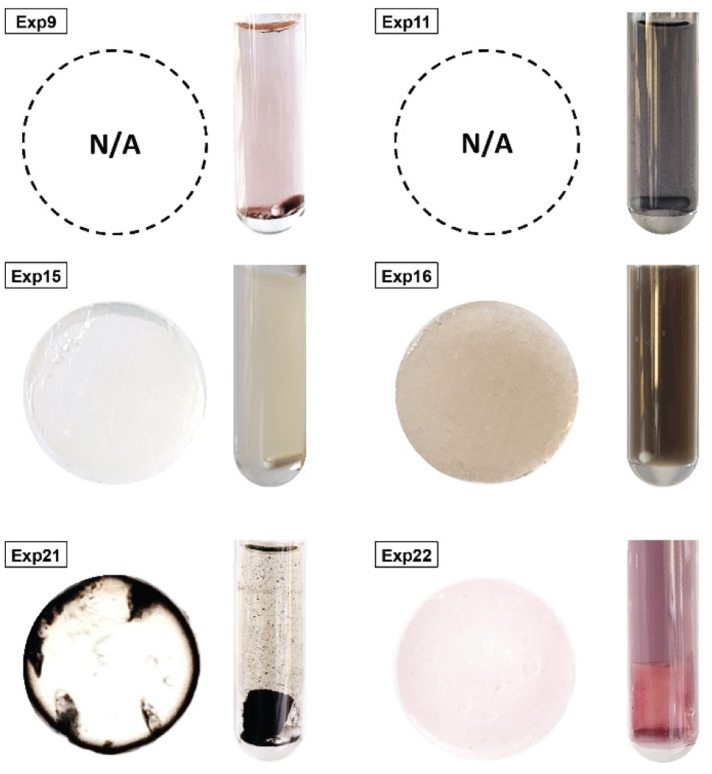
Experiments identified as outliers showcasing a cross-sectional view of the hydrogels (if formed, which was not the case for Exp 9 and Exp 11) alongside a photograph of the microwave reaction vessel. Reaction conditions can be found in Table S1† with the experiment number.

Since outliers may not always be problematic (*e.g.* some are caused by natural variations), further examination of the outcomes from these six experiments revealed that their exclusion can be well justified. The following observations (see Fig. 4) explain deviation from the corresponding regressions and subsequently confirm their removal: experiments 9 and 11 yielded particles that agglomerated and precipitated at the bottom of the vial alongside the lack of proper gelification for the agarose. Exp 15 showed barely any coloration while similar samples were vibrantly colored. Consequently, there was only a small extent of reduction of the AgNO_3_ precursor. The solution of Exp 16 was colored and led to gel formation but yielded an unexpected black color instead of the typical yellow color for AgNPs. Interestingly, note the obtained circularity (*circ* = 0.69) was far away from the other experiments, which is in principle quite pertinent for our objective of finding reaction parameters that lead to anisotropic morphologies. Nonetheless, Exp 16 must be treated as an outlier and cannot be used for the multivariate regression. Exp 21 displayed a high amount of broken hydrogel pieces. Consequently, the NP formation did not occur at the surface and within an intact hydrogel monolith as for all other non-outlier experiments. Finally, Exp 22 yielded NP formation mainly in the aqueous solution surrounding the hydrogel and not in the volume of the hydrogel itself.

To confirm or infirm the removal of the outliers after the previous analysis, the standardized residuals were calculated (eqn (1)).1
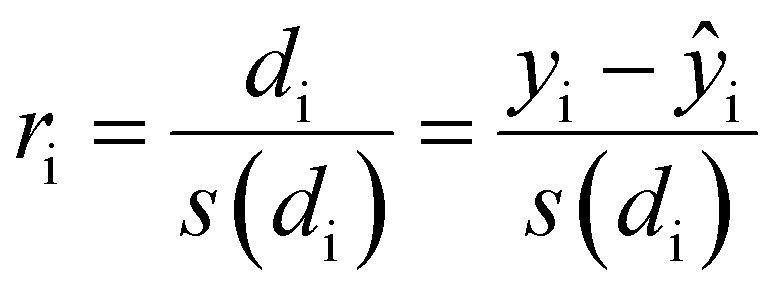
*r*_i_ is the standardized residuals, *d*_i_ is the residual, which is calculated by subtracting the computed response *ŷ*_i_ from the observed response *y*_i_ and *s* is the estimated sample standard variation.

**Fig. 5:**
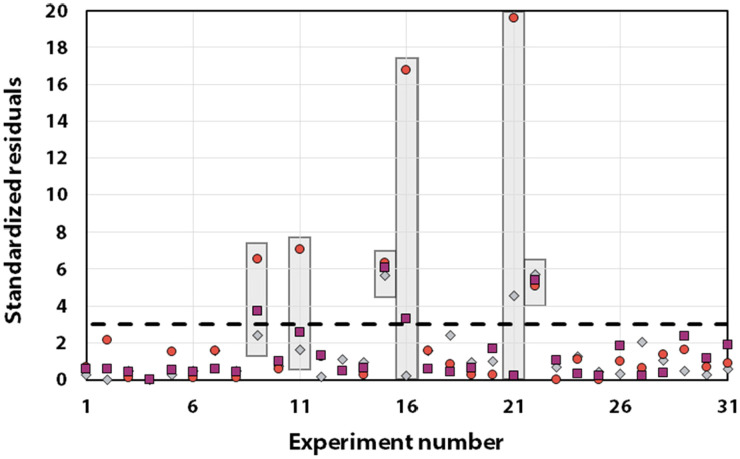
Graph of the standardized residuals measured for all studied responses: diameter (grey diamonds), *circ* (red circles) and *PDI* (purple squares). The outliers’ responses are highlighted in grey boxes. Note that only the data considered as *outliers* exhibit values above the dashed line.

It is usually recognized that a standardized residual (normalized by the standard variation) with a value higher than 3 can be considered an outlier.^58^ This supports our manual expert interpretation-based choice to remove the experiments 9, 11, 15, 16, 21, and 22, since they all have at least one response (mostly *circ*), above the limit value of 3 (see Fig. 5, represented as a dashed black line).

After removal of the outliers and acceptance of the modified model (see the predicted *vs.* actual responses after removal of the outliers’ graphs, Fig. S7†), we next performed an analysis of variance (ANOVA). This includes calculating the probability value (*p*-value) and the coefficient of determination (*R*^2^), which give important information about the model's relevance (see Table S2†). A *p*-value < 5% (in order of significance: PDI*, followed by D** then circ***) indicates that a significant relationship is described by the model (the level of significance is shown as asterisks, where *, ** and *** corresponds respectively to *x*%, 0.*x*% and 0.0*x*% levels). Since we obtain low *p*-values of 0.115, 0.028, and 1.869% for *D*, *circ*, and *PDI* respectively, it is possible to reject the null-hypothesis, *i.e.*, no relationship between the variables (*X*_*n*_) and the responses (*Y*_*n*_). In addition, *R*^2^ gives information about how much the variability can be explained by the model (0 being no variability can be explained by the model and 1 meaning all variability is explained by the model). In our case, both *D* and *circ* regressions significantly explain the variance of the response (*R*^2^ > 0.9), while the *PDI* model is less significant, but still displays good statistical relevance (*R*^2^ > 0.8). It is noteworthy that the *R*^2^ were improved by the removal of the outliers from the initial regression (containing the outliers) with 0.790 for *D*, 0.577 for *circ* and 0.604 for the *PDI* to respectively 0.918, 0.939 and 0.844. The chosen morphological descriptors result from complex phenomena (nucleation and growth, followed by aging mechanisms such as coalescence and Ostwald ripening).^54^ Accounting for all variability (described by *R*^2^) using only basic predictors such as temperature and concentration is not sufficient to describe the complexity. Thus, given the complexity of the morphology underlying phenomena, the obtained regressions are surprisingly good.

In order to check the initial assumption of the lack of interaction between the regression variables, we analyzed the variance inflation factors (VIF), which are measures of how much collinearity exists between each regression coefficient (*b*_*n*_) and consequently each variable (*X*_*n*_). Collinearity is a situation where the predictors are correlated with each other, which should not be (predictors are supposed to be independent variables) – high collinearity might hinder model interpretation, stability, and efficiency. These factors are presented in detail in the ESI (see Table S3†). In essence, if a VIF is above 5, a high level of collinearity between the effects can be expected, while a value above 10 indicates problematic collinearity.^59,60^ Due to inconsistent results of the VIF for each individual level when we have categorical predictors (the VIF assumes that the predictors are continuous and have a linear relationship with the response variable), we are rather interested in the variance factor of the categorical predictor as a single entity, which is known as the generalized VIF (GVIF) and is presented in Table S4.† More specifically, Fox and Monette recommended using the adjusted generalized standard error inflation factor (aGSIF) for categorical predictors with more than two levels, as it adjusts for the number of levels and allows comparability with other predictors (Table S4†).^61^ In this case, values of aGSIF superior to √2.5 (1.6) may be of concern and highlight significant collinearity while values above √5 (2.2) or √10 (3.2) are indicative of a more severe issue in collinearity and would be considered unnaceptable.^61^ In our case, this means that there is a significant level of interaction between *b*_1_, *b*_2_ and *b*_4_ (synthesis temperature, time and heating rate) since their aGSIF are slightly above 1.6 (1.60, 1.84 and 1.89, respectively) while not being a critical matter. This makes sense because these factors impact one another. Indeed, heating at 5 °C min^−1^*versus* 20 °C min^−1^ means that it takes four times longer to heat to a given *T*, which generates markedly different total experimental times (heating time plus reaction time *t*_h_ + *t*_R_). For example, for 5 min of synthesis at 100 °C with a heating rate of 5 °C min^−1^, total time is 21 min = *t*_h_ + *t*_R_*vs.* 9 min = *t*_h_ + *t*_R_ for a heating rate of 20 °C min^−1^, which is a significant difference. Furthermore, using these same two conditions for temperature, we can calculate a noticeable different average temperature compared to the desired synthesis temperature: an average synthesis temperature of 69.5 °C is found for a heating rate of 5 °C min^−1^, while 82.2 °C is found for the 20 °C min^−1^ heating rate. To address co-linearity, two remedies are possible: (i) one of the highly correlated variables could be removed, since the information retrieved from this variable is considered redundant when high collinearity exists, or (ii) one could combine these input variables into a new one that would properly showcase the contribution of each variable (*e.g.*, total synthesis time (*t*_tot_ = *t*_r_ + *t*_h_) and average synthesis temperature *T̄*). In addition to aGSIF, using the adjusted *R*^2^ (to account for overfitting) would help decide between the necessary predictors needed in a further study.

Before analyzing the model, we shall inspect the responses’ graphs for any trends. The experimental diameter was plotted against circularity (Fig. S8†) and against *PDI* (Fig. S9†) in a set of graphs representing the different levels (colour coded) of a specific factor. Note that a population is usually considered monodisperse when the *PDI* is 0.1 or less (represented with a dashed line). First, irrespective of the factors, the circularity tends to be lower with increasing size (see the downward trend in Fig. S10†). Although the *R*^2^ value of this trend is 0.328, it is important to note that this observation does not imply that the two variables are not independent as required and does not suggest co-dependency between them. We did not find any studies dealing with nanoparticles size that take both changes in size and circularity into account.

Another observable trend concerns the three factors that have some level of collinearity (*X*_1_, *X*_2_ and *X*_4_) with respect to circularity (Fig. S8A, B and D†). It seems that lower temperatures (100 and 120 °C), longer reaction times (30 min and 180 min), and slower heating (5 °C min^−1^) yields on average more circular particles. On the contrary, higher temperatures (150 and 180 °C), shorter reaction times (1 min and 5 min) and faster heating (as fast as possible, AFAP) yields reduced circularity in average. This would make sense since a quicker synthesis can be achieved at higher temperature, lower reaction time and/or faster heating rate, and *vice versa* for the opposite conditions. A slower reaction would give time for the particles to express near-spherical morphology (in reality, for the crystallinity of metal NPs, polyhedral particles with high numbers of vertices) through the successive consumption of primarily the highest surface free energy facets. Consequently, circularity would increase for slower reactions, while faster ones would rather lead to out-of-equilibrium shapes (*i.e.* circularity being further away from 1). Another interesting trend concerns the choice of metal precursor. In order to improve their visualisation, Fig. S8E and S9E,† were combined into a three-dimensional representation (Fig. S11†). In this graph, it can be clearly seen that experiments using the Na_2_PdCl_4_ as a precursor displays similar diameters and *PDI*s while circularity varies independently.

**Fig. 6:**
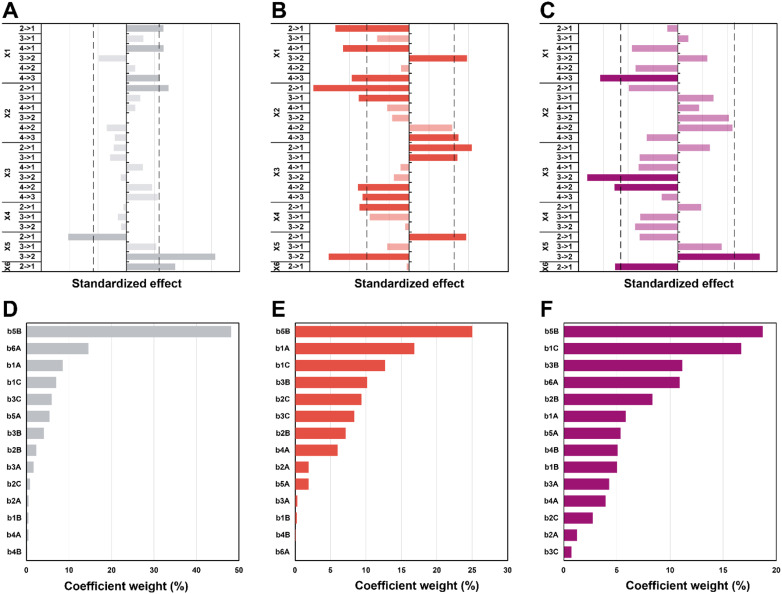
Effects graphs for *D* (A), *circ* (B) and *PDI* (C). The dotted line corresponding to a confidence interval of 95%, indicating a statistically significant effect. The effects which are not significant are faded. Coefficient graphs representing the weight each coefficient has on the variation of the diameter (D), circularity (E) and *PDI* (F).

**Scheme 2:**
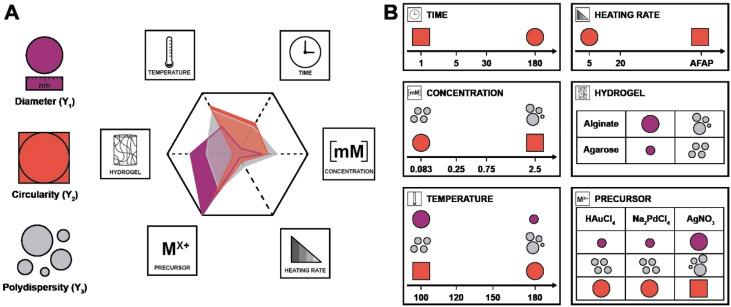
Summary of the factors’ weights (A) and effects (B) on each of the responses (in purple, *D*, red, *circ* and grey, *PDI*). For each factor except the choice of precursor (presented as the change to AgNO_3_), the effects are presented for an increase in level (*e.g.* when the temperature is increased from level 1 to 4, the diameter diminishes).

In the first set of 3 graphs, we can see the effects graphs (Fig. 6A–C). These graphs show the effect when a specific level (see Table 1 for the levels) is changed to another one for a same factor (*e.g.* 3 → 1 for *X*_1_ means that the temperature goes from level 3 = 150 °C to level 1 = 100 °C). In the second set of 3 graphs (Fig. 6D–F), it is possible to observe the Pareto graphs of each coefficient (see Table S3† for the coefficient values *b*_*n*_) which gives the dominant contributors to the variability in the different responses and is useful to assess the relative contribution of each factor (normalized to give 100%). For the diameter (Fig. 6A and D), a significant effect can be seen going from high synthesis temperature (*X*_1_) to lower temperatures (180 to 150 and 100 °C plus 120 to 100 °C; respectively 4 → 3, 4 → 1 and 2 → 1). It seems that in the hydrothermal regime, we tend to have smaller particles as compared to the boiling point – in the literature, an increase in temperature is commonly associated to a decrease in size.^62,63^ Then, a significant effect (larger diameter) is observed only from 5 min to 1 min (2 → 1) of synthesis time (*X*_2_), which is intriguing since no other significant effect can be observed for this variable – this must be linked to the high collinearity of this level since 1 min is quite short and will be more subject to outcomes from temperature and heating (distorting the true effect of time). Next, for the choice of precursor (*X*_5_), a great effect can be observed when changing Na_2_PdCl_4_ back to AgNO_3_ (3 → 2), which points towards a significantly larger diameter. Finally, going from agarose to alginate (2 → 1 for *X*_6_), it is possible to observe an increase in diameter. This makes sense, since the synthesis goes from a synthesis in the whole solution (with consequently a high number of nuclei) to a growth restricted to the interface of the alginate hydrogel with the surrounding aqueous solution. Limited initial nuclei for a certain supersaturation level combined with a continuous feed of additional metallic ions will favor growth, not further nuclei formation (*i.e.* increasing *D*). In order of importance, the coefficients (see eqn (S3);†*b*_0_ corresponds to the level 1 for all the factor while *A*, *B*, and *C* correspond respectively to levels 2, 3, and 4) that have the most impact on diameter are the following (see Scheme 2): the choice of metallic precursor (*b*_5A_ and *b*_5B_), the choice of support (*b*_6A_), the synthesis temperature (*b*_1A_ and *b*_1C_), and the synthesis concentration (*b*_3B_ and *b*_3C_).

Moreover, effects over the circularity were studied (Fig. 6B and E), with a first impression that a lot of parameters statistically affect this response. Overall, decreasing temperatures (*X*_1_) seem to statistically decrease circularity, with a singular opposite effect when going from 150 °C to 120 °C (3 → 2) which increases it instead. Typically, it is known that increasing temperature enhances the nucleation rate, but strongly depresses growth rates.^62,64^ A higher number of nucleation centres would subsequently result in more perfect crystals leading to higher values of circularity. Yang and Pan have noticed that alginate acts as a capping agent and adsorbs preferentially on (111) planes of Ag nuclei – this is enabled at temperatures of *ca.* 120–180 °C.^30^ While this is true for AgNO_3_, it might not happen for HAuCl_4_ and Na_2_PdCl_4_. Furthermore, while temperature might enable the anisotropic growth, not having sufficient time to grow anisotropic particles for example might inhibit this phenomenon (the syntheses of Yang and Pan lasts for 6 h). Again, we see here why temperature has a high value of VIF. The next two parameters, synthesis time (*X*_2_), and concentration (*X*_3_), both display a similar pattern. For a higher time (180 min to 30 min; 4 → 3) and lower concentrations (0.75 mM and 0.25 mM to 0.08 mM; 3 → 1 and 2 → 1), it is possible to see a negative impact on circularity, while for lower time (30 min and 5 min to 1 min; 3 → 1 and 2 → 1) and higher concentrations (2.5 mM to 0.75 mM and 0.25 mM; 4 → 2 and 3 → 2), the opposite is seen (*i.e.*, *circ* increases). Indeed, one can expect that the drop in circularity is caused by the lower number of nuclei formed and more time in the growth phase, where the particles have the time to increase in anisotropy, as explained beforehand. The reasoning is valid for the opposite phenomena, where a higher concentration and lower synthesis time would force the system to be in supersaturation forming more nuclei and less time for these nuclei to move away from the circular shape. Next, an unexpected effect with respect to the heating rate (*X*_4_) is observed: going from 20 °C min^−1^ to AFAP (2 → 1) increases the circularity. While this is in line with what was expected at first, the associated weight is quite low (5%), which, when added to collinearity, leads to the conclusion that the heating rate is not as relevant as we expected at first. Again, for the choice of precursor (*X*_5_), going from palladium to silver (2 → 1) increases *circ*. Put together, this means circularity tends to be lower for silver (displays more anisotropy). Then, an interesting effect is the choice of support (*X*_6_): it has absolutely no impact whatsoever on circularity. This is quite interesting for two reasons: the choice of alginate or agarose as the polysaccharide, plus the synthesis at the interface or in the volume both do not matter in the end for circularity. For the coefficient weights (see Scheme 2), the choice of precursor (*b*_5B_) – and consequently the type of metal NPs formed – has the most impact, followed by temperature (*b*_1A_ and *b*_1C_), concentration (*b*_3B_ and *b*_3C_), and time (*b*_2B_ and *b*_2C_).

Lastly, the effects that are statistically relevant for *PDI* are presented (Fig. 6C and F). Going from 180 °C to 150 °C (4 → 3 for *X*_1_) has a strong negative effect on *PDI* – the reaction might proceed too fast at high temperature, yielding many different sizes, not providing sufficient time for the classic nucleation and growth expectation. A parallel can be drawn with NaBH_4_ (a strong reducing agent) reducing a metal precursor, leading to a rapid formation of small nuclei, followed by coalescence, which tends to induce polydispersity.^65,66^ Then, an interesting effect concerns concentration (*X*_3_): changing 2.5 and 0.75 mM to 0.25 mM (4 → 2 and 3 → 2) has a significant impact towards monodispersity. The two highest concentrations increase saturation and more nuclei are expected to be formed over time, generating a more size-dispersed NPs population.^55^ As observed previously, changing the precursor to AgNO_3_ (3 → 2 for *X*_5_) has a strong effect on the response – the silver precursor tends to favor higher polydispersity. Interestingly, while the choice of support has no effect at all on the circularity, the NPs get more polydisperse in alginate (2 → 1 for *X*_6_) – it makes sense that a homogeneous synthesis in solution would lead to a narrower NP population as opposed to a heterogenous synthesis limited to the gel surface. Interestingly, weights for the *PDI* coefficients are more evenly distributed for the different factors (see Scheme 2).

**Fig. 7:**
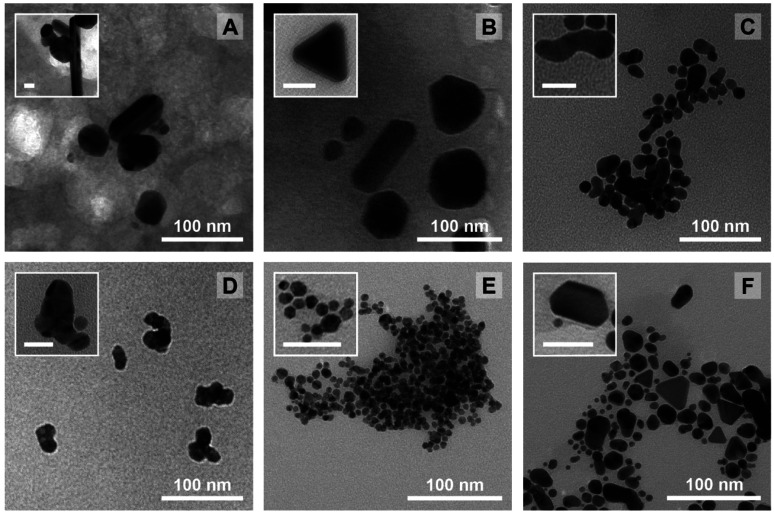
TEM micrographs for experiments that yielded out of the ordinary NPs alongside high resolution TEM micrographs in the insets (the scalebar represents 25 nm). (A) Rod-like Ag particles from Exp 6. (B) Highly angular Ag particles from Exp 8. (C) Fused Au NPs in Exp 12. (D) Bizarre, coalesced particle for Exp 16. (E) Small PdNPs showcasing the highest degree of angularity for all generated PdNPs in Exp 21. (F) Exp 23 AuNPs showcasing interesting shapes such as triangular or irregular hexagonal platelets.

Again, it needs to be said that this work is exploratory: we wanted to understand which factors are the most crucial to control the three most important parameters defining NP morphology (*i.e.* diameter, circularity and polydispersity index). All these results are summarized in Scheme 2. On the left side, each response is schematised with their associated colour (from top to bottom: *D* in purple, *circ* in red, and *PDI* in grey) and a radar plot shows the importance of the factors *X*_*n*_ over the responses *Y*_*n*_ (the middle represents no effect while the periphery is the biggest effect). On the right side, statistically relevant parameters that induce a change in *D*, *circ* and *PDI* are represented alongside the change in level. Finally, the most interesting particles are displayed in Fig. 7 and their macroscopic aspect in Fig. S12.† Our goal was to produce particles that had interesting characteristics. In the end, 6 out of 31 experiments were able to achieve this purpose. Taking the experiments 6 and 8 (Fig. 7A and B), these NPs have interesting geometrical shapes such as rods for Exp 6 and angular shapes such as triangles for Exp 8. This seemed to emerge when the support is Ca-alginate and the precursor is AgNO_3_. For experiment 12 (Fig. 7C), high heating rate and high temperature seemed to have led to the partial coalescence of the particles which are quite stable (the agarose did not gellify properly). While Exp 16 and 21 (Fig. 7D and E) were discarded from the model (as identified as outliers), it is still noteworthy to highlight their striking characteristics. In contrast to Exp 6 and 8, Exp 16 was done in agarose, at higher concentration and 150 °C, which favored the formation of these ill-defined particles. Concerning Exp 21, the highest angularity for Pd^0^ NPs was observed, which is highly interesting, since the other PdNPs tend to be relatively small, spherical and monodisperse. In this experiment, the high concentration of Pd precursor led to these higher angularity particles with still impressive monodispersity. As compared to all AuNP@hydrogel samples, Exp 23 favoured out-of-equilibrium shapes, because of the higher temperature and heating rates employed.

## Conclusions

In conclusion, this work presents a new green and facile approach to synthesize supported noble metal nanoparticles in hydrogels by hydrothermal synthesis. This synthesis was realized under various hydrothermal conditions (120, 150 and 180 °C) and requires solely a mild solvent (water), a biopolymer (agarose or Ca-alginate) and a metallic precursor. The polysaccharides play the triple role of both the reducing agent, the support and the chemical anchor groups connecting the NPs to the support. Hence no additional conventionally employed reductants or anchoring chemicals (both often toxic and environmentally harmful) are needed. Full conversion to the metal NPs@hydrogel materials can be achieved under these conditions. The choice of hydrogel allows control over spatial localization of NPs in the hydrogel – an uncommon feat in the literature. Yoon *et al.* have synthesized AuNPs spatially in PVOH hydrogels thanks to a controlled diffusion enabling their tailored synthesis in distinct areas of the hydrogel matrix (*i.e.* precise manipulation of the size and morphology of NPs).^53^

Gisbert Quilis *et al.* strategically localized NPs at the hydrogel surface to facilitate the formation of collective localized surface plasmons within a lattice structure – advantageous for advanced sensing and photonic applications.^67^ These materials could also be of interest as flow-through catalytic reactors,^68^ in biomedical applications,^69^ such as tackling antibacterial resistance,^25^ or stimuli-responsive and switchable conductive composites.^70^ In addition, an attempted DoE study remains unfinished, underpinning the difficulty of using sensitive media (*i.e.* natural hydrogels) that have limited resistance to hydrothermal conditions. However, the results could still be exploited with a statistical study by a multivariate regression fit under the assumption that there is no interaction between the studied factors. This regression was exploited to identify trends to look for in nanoparticles synthesis in order to thoroughly control the final morphology – to our knowledge, this is a first for the synthesis of metallic colloids. The heating rate was the only parameter with little effect on the different responses and in hindsight could have been discarded due to its high level of collinearity. However, for a predictive study, it might be important to monitor the heating rate in detail, since it does bring some information that would add to the quality of the overall prediction through more input data. Through this study, several interesting morphologies for all three types of metallic NPs (Au, Ag, and Pd) could be observed, thereby meeting the initial goal of this study. In a future work, it would be worthwhile to explore the use of various precursors of the same metal (*e.g.* different Pd salts) for hydrothermal synthesis.

## Data availability

In accordance with the principles of transparency and reproducibility, we have included the curated data used for statistical analysis in this research in the ESI of this article (in ESI Tables S1 through S4†). Researchers can access and utilize this data to independently perform the statistical analyses. Additionally, the software employed for experimental design is Azurad®, and additional statistical analyses were conducted using R, a free software for statistical computing available online.

## Author contributions

Olivier Gazil: Conceptualization, methodology, investigation, data curation, formal analysis, writing – original draft, visualization. Daniel Alonso Cerrón Infantes: Visualization. Nick Virgilio: Conceptualization, funding acquisition, writing – original draft, supervision. Miriam M. Unterlass: Conceptualization, writing – original draft, funding acquisition, resources, supervision.

## Conflicts of interest

There are no conflicts to declare.

## Supplementary Material

NR-016-D4NR00581C-s001
