# Supplementary material for: Hydrothermal synthesis of metal nanoparticles@hydrogels and statistical evaluation of reaction conditions’ effects on nanoparticle morphologies
Source: Nanoscale. 2024 Sep 6;16(38):17778–92. doi: 10.1039/d4nr00581c (PMC11377975; doi:10.1039/d4nr00581c)
Supplement: NR-016-D4NR00581C-s001 [file NR-016-D4NR00581C-s001.pdf]

Supplementary Information (SI) for Nanoscale.
This journal is © The Royal Society of Chemistry 2024

Supporting information

Hydrothermal Synthesis of Metal Nanoparticles@Hydrogels and Statistical Evaluation of Reaction Conditions on Nanoparticle Morphologies

Olivier Gazil,[a,b] Nick Virgilio [b] and Miriam M. Unterlass *[a,c]

[a.] Universität Konstanz, Department of Chemistry, Solid State Chemistry, Universitätsstrasse 10, D-78464 Konstanz, Germany. E-mail: miriam.unterlass@uni-konstanz.de.

[b.] CREPEC, Department of Chemical Engineering, Polytechnique Montréal, C.P. 6079 Succursale Centre-Ville, Montréal, Québec H3C 3A7, Canada

[c.] Center for Molecular Medicine of the Austrian Academy of Sciences (CeMM), Lazarettgasse 14, AKH BT25.3, 1090 Vienna, Austria

I. Methodology

II. Supplementary Figures

III. Supplementary Tables

IV. Supplementary Equations

V. References

I. Methodology

Materials

Hydrogen tetrachloroaurate(III) trihydrate ($HAuCl_4 \cdot 3H_2O$), a high melting point (~88 °C) agarose and a low viscosity (20 cps at 20 °C and 1 % w/v) alginic acid sodium salt (SA) were purchased from Alfa Aesar (reference numbers 36400, J61123 and B25266, respectively). Silver nitrate ($AgNO_3$), sodium tetrachloropalladate(II) (Na_2PdCl_4), and glucono delta-lactone (GDL) were all purchased from Sigma-Aldrich (reference numbers 792276, 205818, G2164). Sodium citrate, calcium carbonate ($CaCO_3$) and calcium chloride ($CaCl_2$) were all gathered from the university chemical warehouse ("chemikalienlager") and came from Alfa Aesar. All chemicals were of reagent grade and were used as received without further purification.

Preparation of sodium alginate hydrogels

1 cm^3 alginate hydrogel cylinders (0.5 cm radius x 1.3 cm height) were prepared by the internal setting method as described by Draget et al. with $CaCO_3$ and GDL.[1] Briefly, 2% w/v SA was dissolved in water at 80 °C, followed by the addition $CaCO_3$ (30 mM), resulting in a homogeneous suspension. Then, a GDL solution (60 mM) was freshly prepared and quickly added to the SA/$CaCO_3$ suspension, which was then poured in a polylactide 3D printed mold. After overnight gelification, the gels were plunged in 2% w/v $CaCl_2$ solution to finalize crosslinking.

Preparation of the metal nanoparticles (NPs) @ alginate and @ agarose hybrids

Each SA cylinder was plunged into 4 mL of an aqueous metallic precursor ($HAuCl_4$, $AgNO_3$ or Na_2PdCl_4) solution (0.083 mM, 0.25 mM, 0.75 mM or 2.5 mM) in a glass vial inside a steel autoclave. To use a faster heating rate, some solutions and samples were added to G10 glass vials for Monowave© microwave reactor. The vials were heated in an Anton Paar Monowave 450 microwave reactor at a chosen temperature (100 °C, 120 °C, 150 °C or 180 °C) and heating rate (as fast as possible – AFAP, 20 °C/min or 5 °C/min).

For agarose hydrogels, 5 mL of 2% w/v agarose was dissolved in 80°C water followed by the addition a concentrated solution of metallic precursor (10 mM) to yield the desired concentration in the hydrogel solution (0.083 mM, 0.25 mM, 0.75 mM or 2.5 mM). Then, the suspensions were added either into glass vials in the autoclave, or into G10 glass vials for the desired hydrothermal synthesis.

After the syntheses, the resulting NPs@alginate are directly recovered while the NPs@agarose are melted and poured into the 3D printed cylindrical mold.

Optical microscopy characterization

Observations were performed with a reflected light microscope (Leica M125 optical microscope) at 4X magnification.

Transmission electron microscopy (TEM) characterization

Two different techniques were used for the observation of the nanoparticles. The first one required the solubilization of the hydrogel to cast a droplet on a TEM copper grid (400 mesh with 10-12 nm carbon film), followed by wicking to remove excess liquid from the drop. For the NPs@alginate, 0.1 mL was added to 0.9 mL of citrate solution (50 mM) which allows chelation of the Ca^{2+} ions and solubilisation of the gel. As for the NPs@agarose, the gel was melted at 85°C and 0.1 mL was added to 0.9 mL of deionized water at 85°C.

The second preparation technique required the immobilization of the gel in epoxy, followed by ultramicrotomy (~100 nm thickness) at room temperature and deposition of the thin slices on TEM

grids. Samples were prepared first by gradually dehydrating the gel with ethanol, which is then replaced by Spurr's resin, as described by Yoon et al.[2]

TEM images were obtained with two different instruments: a Libra120 EF-TEM instrument and a FEI Tecnai F20 S/TEM equipped with a GATAN Rio-16 camera. The observations were realized respectively at 120 kV and 200 kV.

Nanoparticles morphological descriptors

NPs diameters *D*, circularity *circ* and polydispersity index *PDI* were determined by analyzing 100+ particles with the image analysis software ImageJ. *D* is defined as a segment that would pass through the middle of an equivalent area circle, while *circ* is a measure of how round and smooth a shape is (1: perfect circular shape; 0: highly non-circular shape) in 2D digital image analysis (Eqn. S1). The *PDI* is a measure of the heterogeneity of a population distribution (Eqn. S2).

II. Supplementary Figures

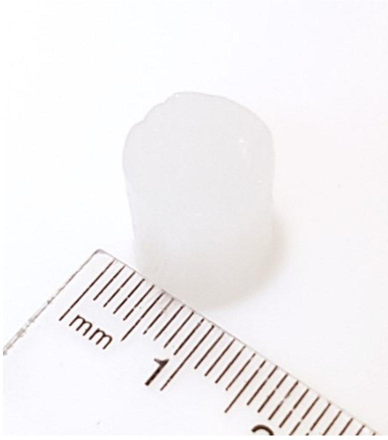

Figure S1. Macroscopic aspect of a pristine calcium alginate hydrogel cylinder before NPs synthesis.

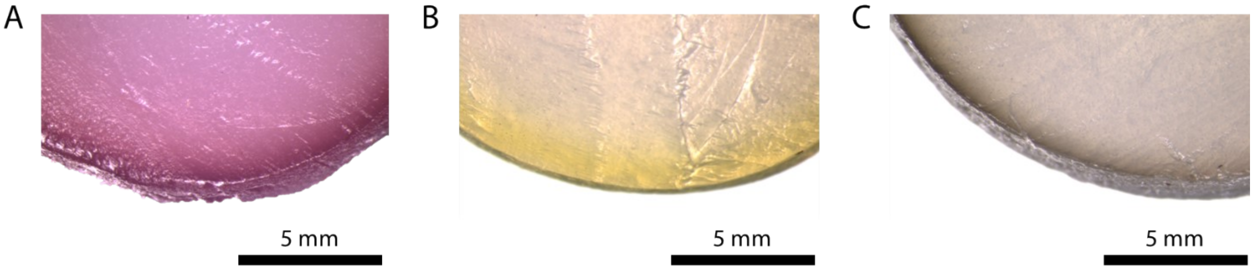

Figure S2. Optical micrographs of the samples presented in Fig.1A-C at higher magnification: A) AuNPs@alginate; B) AgNPs@alginate; C) PdNPs@alginate. Note for all three cases the highly contrasted region near the interface, encompassing the majority of the synthesized NPs.

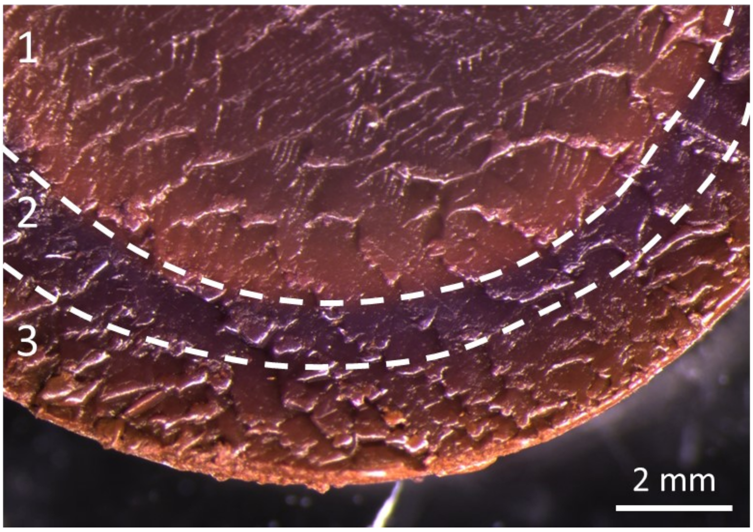

Figure S3. Optical micrograph of AuNP@alginate with reaction conditions of T_R = 120°C, t_R = 3 h and [$HAuCl_4$] = 2.5 mM, highlighting the 3 distinct regions (separated by white dotted lines).

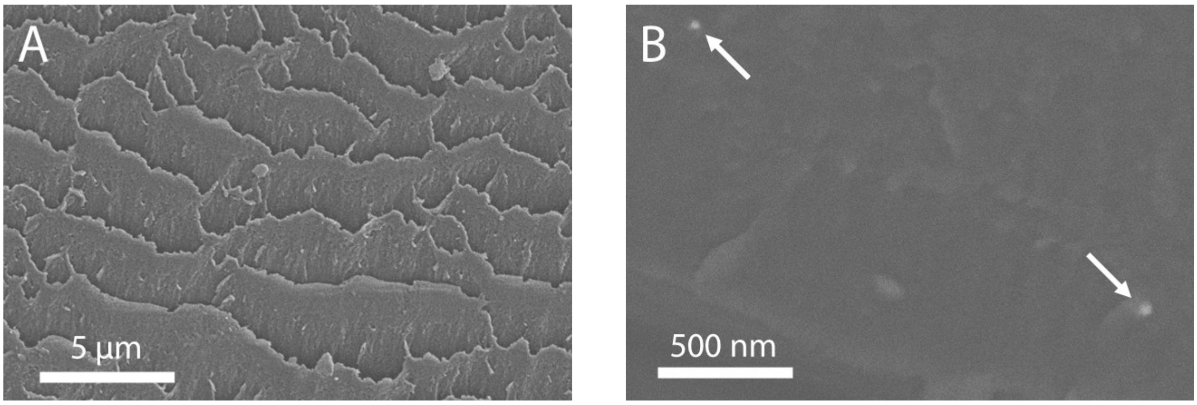

Figure S4. SEM micrograph of: A) a clear alginate hydrogel matrix with no visible gold NPs; and B) a section where two gold nanoparticles can be seen in the hydrogel matrix (indicated by white arrows). In Panel B, the operation is conducted near SEM resolution, which limits the obtained results. Thus, conclusions about selective localization cannot be attained because of the small NPs' sizes.

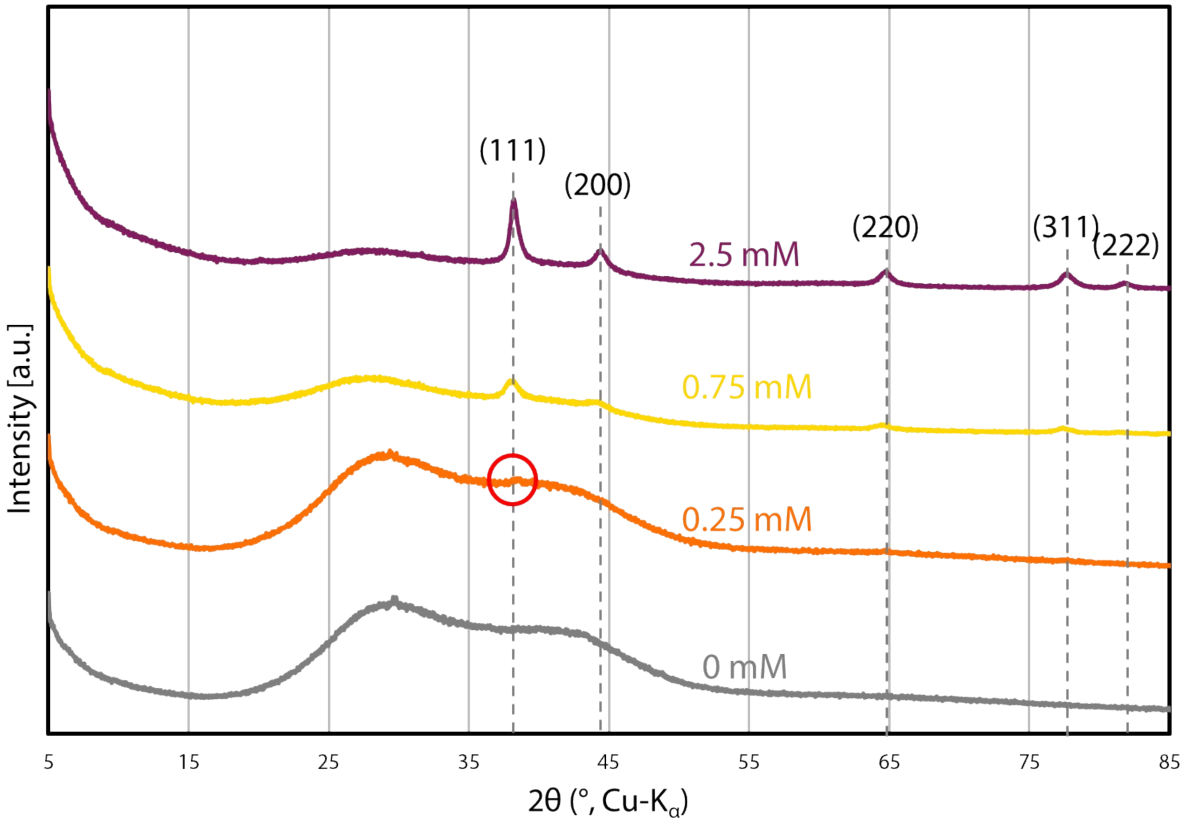

Figure S5. X-ray diffractograms of AuNPs@alginate with varying concentration of metal precursor identified on the curve (0, 0.25, 0.75, and 2.5 mM). The corresponding Au lattice planes with their *hkl* labels. Note the highest reflex of gold (111) barely visible in the 0.25 mM sample. Importantly, all other samples of 0.25 mM and below did not yield any significant results.

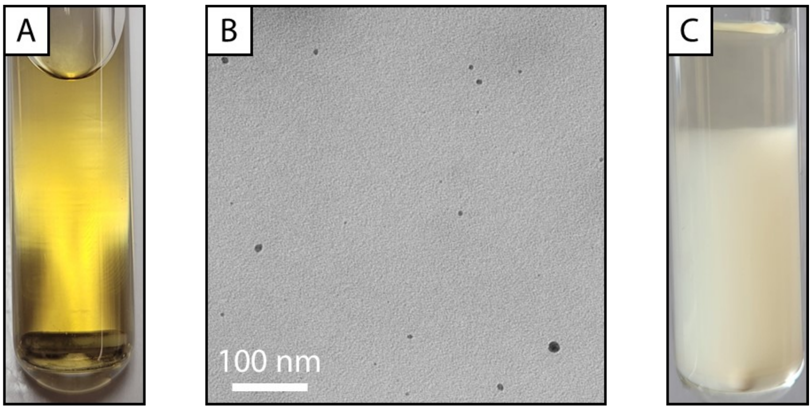

Figure S6. A) Photograph of the microwave reaction vessel containing agarose with the following synthesis conditions: T_R = 180°C, t_R = 1 min, [Ag^+] = 0.75 mM, and heating rate of 5 °C/min. B) TEM micrograph of the resulting solution. C) Photograph of the microwave reaction vessel containing only agarose at the same synthesis conditions as in A, without the silver precursor. Note the degradation of the agarose gel which is precipitated out of solution and sedimented in the vial.

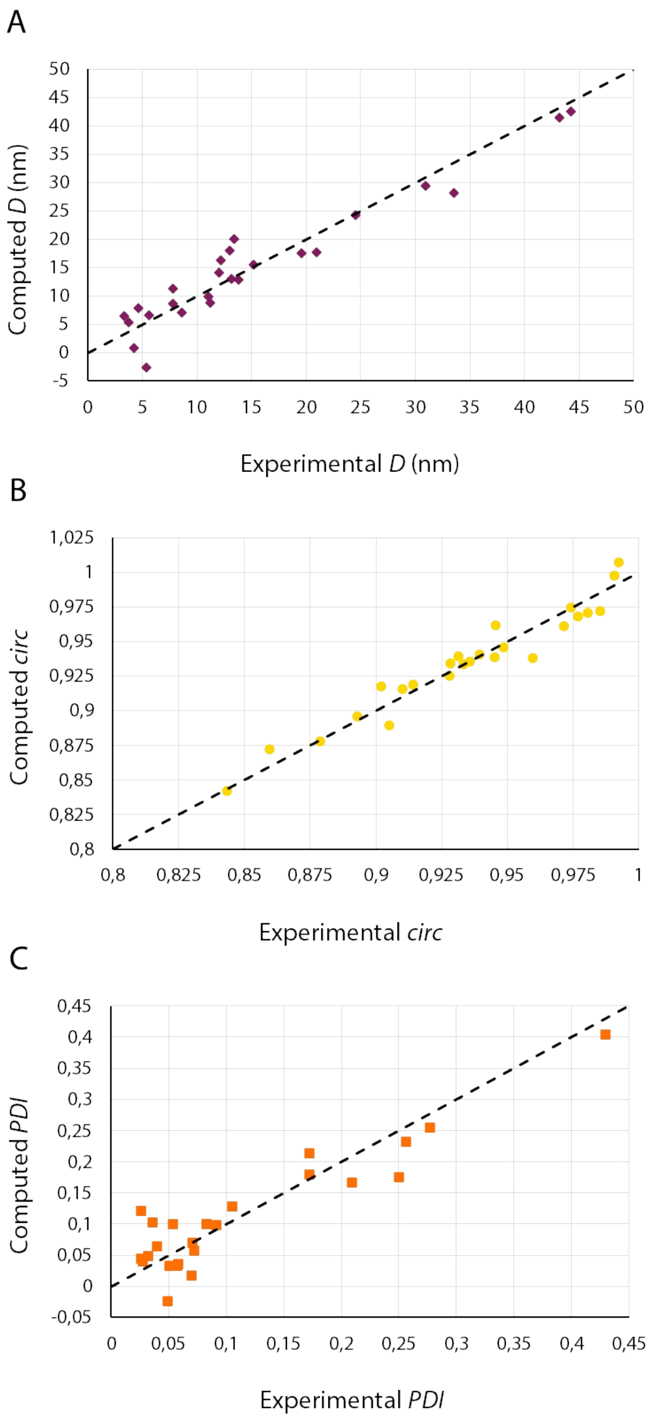

Figure S7. Computed responses (from regression) versus experimental responses after removal of the outliers for the diameter (A), circularity (B) and *PDI* (C), see methodology. The doted line represents ideal prediction by the model (e.g. a computed diameter of 20 nm corresponds perfectly to the observed diameter of 20 nm). Note that the data points are homogeneously distributed around the doted line meaning a strong correlation from the model (i.e. no observable trend indicates a model which cannot be further improved).

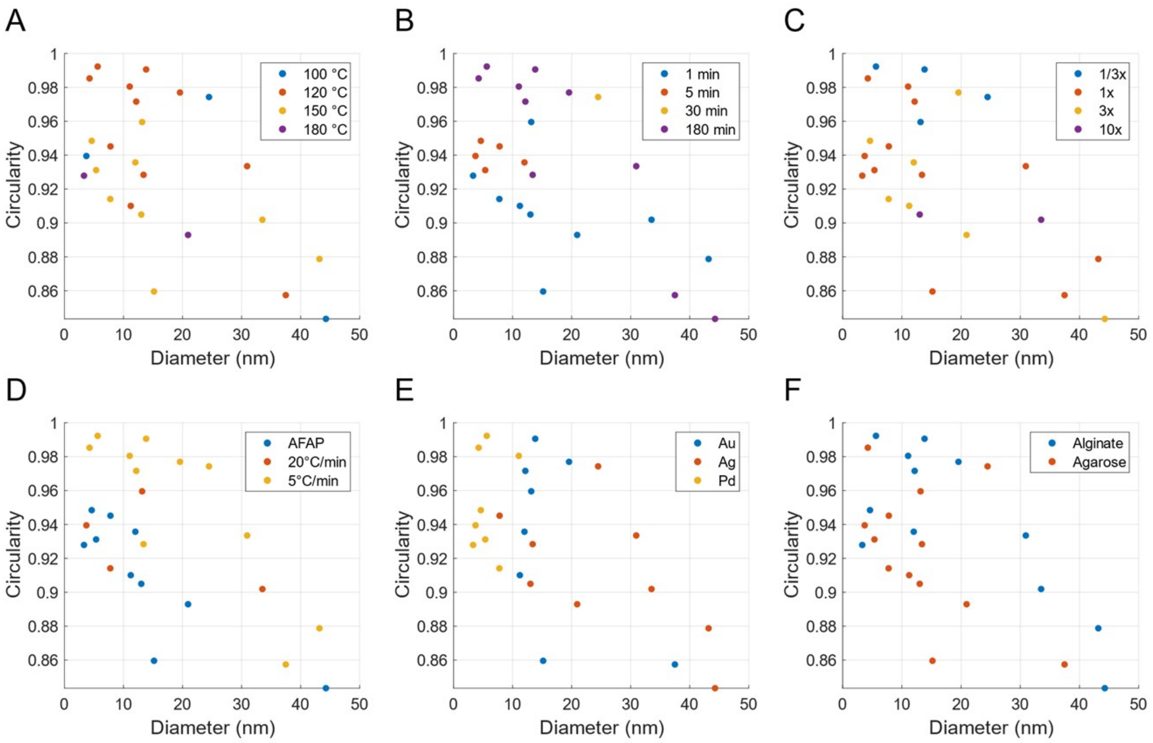

Figure S8. Graphs of the circularity versus the diameter of the nanoparticles with the different factors showcased: synthesis temperature (A), synthesis time (B), precursor concentration (C), heating rate (D), type of precursor (E) and hydrogel choice (F).

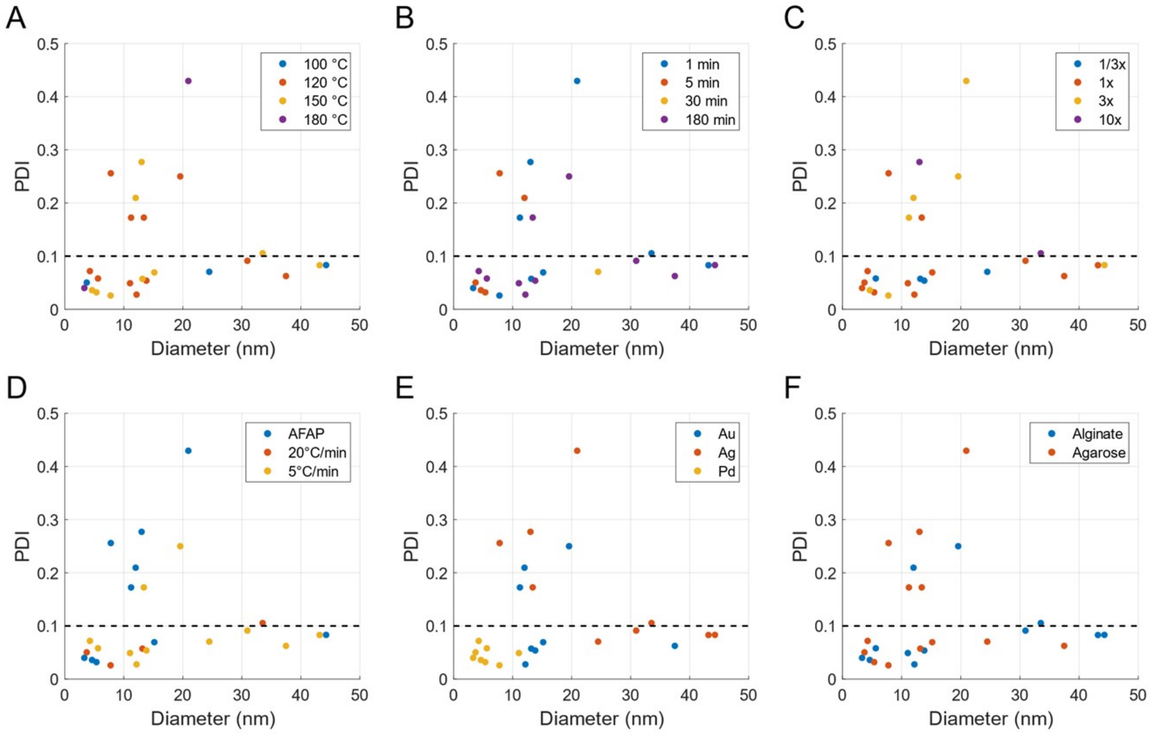

Figure S9. Graphs of the PDI versus the diameter of the nanoparticles with the different factors showcased: synthesis temperature (A), synthesis time (B), precursor concentration (C), heating rate (D), type of precursor (E) and hydrogel choice (F). It is generally accepted that a population is considered monodisperse when the PDI is equal to or less than 0.1 (highlighted in the graphs as a black dashed line).[3]

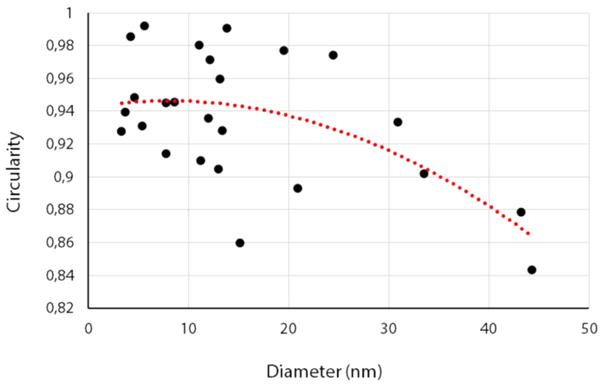

Figure S10. Graph of the circularity versus diameter: note the decreasing trend of circularity with increasing diameter (in red dotted line).

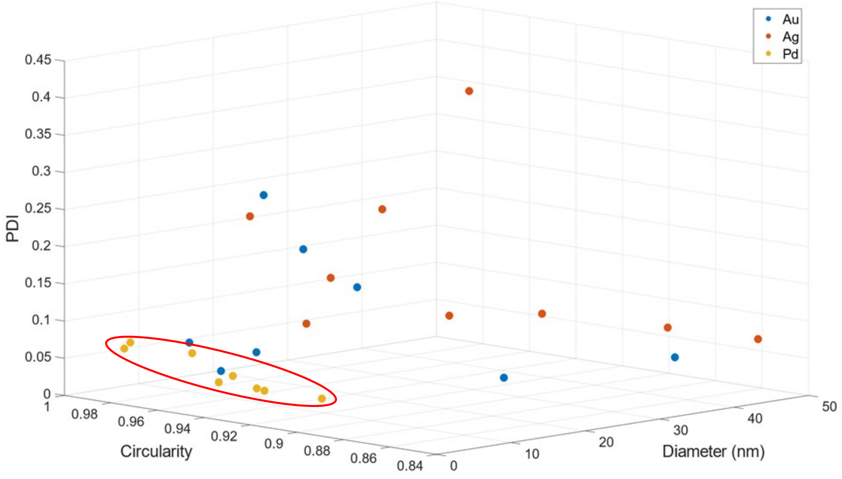

Figure S11. Tridimensional representation of all the responses spaces in respect to the metallic precursor (gold in blue, silver in red and palladium in yellow). Note the bias for the palladium experiments highlighted with a red circle, which displays similar diameters and PDIs, but varying circularities between the experiments.

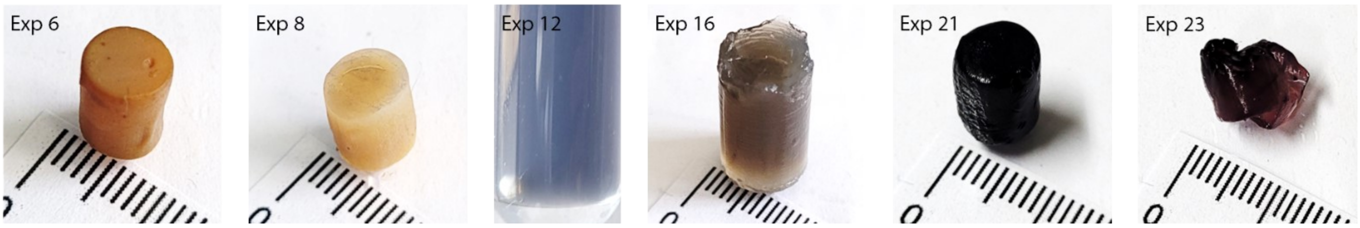

Figure S12. Macroscopic photographs of the experiments that yielded out of the ordinary NPs molded as cylinder (see **Table S1** for reactions parameters). Note Exp 12 which could not be molded after hydrothermal synthesis, and Exp 23 which was fragile and crumbled upon recovery.

III. Supplementary Tables

Table S1. Factors and responses for the experiments that yielded quantifiable results and kept for the statistical analyses.

	Experiment	X_1	X_2	X_3	X_4	X_5	X_6	Y_1	Y_2	Y_3
Successful DoE experiments	**1**	2	2	2	1	2	2	7.8	0.95	0.26
	**2**	3	1	1	2	1	2	13	0.96	0.06
	**3**	1	2	2	2	3	2	3.7	0.94	0.05
	**4**	1	3	1	3	2	2	24	0.97	0.07
	**5**	2	4	1	3	3	1	5.6	0.99	0.06
	**6**	3	1	2	3	2	1	43	0.88	0.08
	**7**	3	1	4	2	2	1	34	0.90	0.11
	**8**	1	4	3	1	2	1	44	0.84	0.08
Randomized experiments	**9**	2	2	3	2	1	2	5.7	0.94	0.11
	**10**	2	1	3	1	1	2	11	0.91	0.17
	**11**	3	1	2	2	1	2	22	0.83	0.08
	**12**	3	1	2	1	1	2	15	0.86	0.07
	**13**	3	1	3	2	3	2	7.8	0.91	0.03
	**14**	4	1	3	1	2	2	21	0.89	0.43
	**15**	3	1	2	2	2	2	11	0.94	0.28
	**16**	3	1	3	1	2	2	31	0.69	0.08
	**17**	3	1	4	1	2	2	13	0.90	0.28
	**18**	3	2	2	1	3	2	5.4	0.93	0.03
	**19**	4	1	2	1	3	1	3.3	0.93	0.04
	**20**	3	2	3	1	3	1	4.6	0.95	0.04
	**21**	2	3	4	3	3	1	7.0	0.89	0.02
	**22**	3	1	2	2	1	1	6.6	0.95	0.11
	**23**	3	2	3	1	1	1	12	0.94	0.21
Initial experiments	**24**	2	4	2	3	1	1	12	0.97	0.03
	**25**	2	4	2	3	2	1	31	0.93	0.09
	**26**	2	4	2	3	3	1	11	0.98	0.05
	**27**	2	4	2	3	2	2	13	0.93	0.17
	**28**	2	4	2	3	3	2	4.2	0.99	0.07
	**29**	2	4	2	3	1	2	37	0.86	0.06
	**30**	2	4	1	3	1	1	14	0.99	0.05
	**31**	2	4	3	3	1	1	20	0.98	0.25

X_1 is the synthesis temperature, X_2 is the synthesis time, X_3 is the precursor concentration, X_4 is the heating rate, X_5 is the metal precursor, X_6 is the hydrogel choice, Y_1 is the diameter (nm), Y_2 is the circularity, and Y_3 is the polydispersity index. Note that the responses Y_1, Y_2 and Y_3 have been rounded in the table for aesthetic purposes but were not when computed in the regression.

Table S2. ANOVA test for the description of particles morphology.

		Squares sum	Degrees of freedom	Mean square	Ratio	p-value (%)	R^2	R_{Adj}^2
**Diameter**	**Regression**	2994.711	14	213.907	7.949	0.115**	0.918	0.802
	**Residuals**	269.109	10	26.911				
	**Total**	3263.820	24					
**Circularit y**	**Regression**	0.03644	14	0.002603	11.048	0.028***	0.939	0.854
	**Residuals**	0.00236	10	0.000235				
	**Total**	0.03880	24					
**PDI**	**Regression**	0.2097	14	0.01498	3.871	1.869*	0.844	0.626
	**Residuals**	0.0387	10	0.00387				
	**Total**	0.2484	24					

Table S3. Coefficient table for the 3 sets of responses alongside the variance inflation factor (VIF).

	Diameter	Circularity	Polydispersity index	VIF
b_0	-13.9 ± 10.4	1.024 ± 0.031	0.367 ± 0.126	-
b_{1A}	19.3 ± 7.3	-0.072 ± 0.022	-0.162 ± 0.088	4.72
b_{1B}	4.0 ± 6.5	-0.008 ± 0.019	-0.132 ± 0.077	10.81
b_{1C}	12.2 ± 5.1	-0.044 ± 0.015	-0.192 ± 0.062	5.98
b_{2A}	3.8 ± 6.0	-0.020 ± 0.018	0.061 ± 0.071	9.194
b_{2B}	-8.1 ± 5.9	0.038 ± 0.017	0.156 ± 0.071	4.737
b_{2C}	-7.6 ± 9.3	0.068 ± 0.027	-0.140 ± 0.111	2.088
b_{3A}	7.2 ± 6.2	-0.008 ± 0.018	-0.117 ± 0.074	3.182
b_{3B}	10.6 ± 5.8	-0.044 ± 0.017	-0.177 ± 0.070	5.738
b_{3C}	11.8 ± 5.4	-0.037 ± 0.016	-0.042 ± 0.064	4.713
b_{4A}	-3.4 ± 5.7	-0.034 ± 0.017	-0.104 ± 0.069	6.889
b_{4B}	-2.6 ± 7.2	-0.004 ± 0.021	-0.149 ± 0.087	6.589
b_{5A}	6.3 ± 3.0	-0.010 ± 0.009	0.064 ± 0.036	1.918
b_{5B}	19.5 ± 3.1	-0.037 ± 0.009	0.123 ± 0.037	1.841
b_{6A}	9.2 ± 2.7	-0.001 ± 0.008	-0.081 ± 0.032	1.533

Table S4. Generalized variance inflation factors (GVIF) for categorical predictors with more than two levels.

	GVIF	DF	aGSIF*
b_1	17.1	3	1.60
b_2	38.3	3	1.84
b_3	4.9	3	1.3
b_4	12.6	2	1.89
b_5	2.2	2	1.22
b_6	1.7	1	1.30

*Adjusted generalized standard error inflation factor (aGSIF); $aGSIF = GVIF^{1/(2*DF)}$, where DF is the degrees of freedom.

IV. Supplementary Equations

$$circ = \frac{4\pi * A}{P^2}$$ Equation S1

circ is the circularity, A is the area of the nanoparticle (nm^2) and *P* is the perimeter (nm).

$$PDI = \left(\frac{D_s}{\bar{D}}\right)^2$$ Equation S2

PDI is the polydispersity index, D_s is the standard deviation of the diameter (nm), and $\bar{D}$ is the average diameter (nm).

$$Y_m = b_0 + b_1X_1 + b_2X_2 + b_3X_3 + b_4X_4 + b_5X_5 + b_6X_6$$ Equation S3

Y_m is the response (diameter, circularity, and polydispersity index), b_0 is the intercept found when each factor is at its highest level, b_n is the coefficient matrix of factor *n* and X_n is the Boolean matrix of the factor *n*. Note that this multivariate regression assumes that there is no interaction between the factors (i.e. their effects are additive).

As an example, we will look at the effect factor 1, *Synthesis temperature* (X_1), has on the diameter for an experiment realised at 120 °C, which is the level denoted as *k* = 1 in the design. We would have the two following matrices for the first factor:

$$b_1 = \begin{bmatrix} b_{1A} \\ b_{1B} \\ b_{1C} \end{bmatrix} = \begin{bmatrix} 19.38 \\ 4.04 \\ 12.24 \end{bmatrix}$$ and $$X_1 = \begin{bmatrix} X_{1A} & X_{1B} & X_{1C} \end{bmatrix} = \begin{bmatrix} 0 & 1 & 0 \end{bmatrix}$$

For an experiment at 120 °C, the coefficient related to this level, b_{1B}, will be the only weighting on the diameter since the Boolean matrix has a value of 1 (true) for the component X_{1B}. In essence, we have *k*-1 binary variables corresponding to the last *k*-1 levels for each of the factors.

$$r_i = \frac{d_i}{s(d_i)} = \frac{y_i - \hat{y}_i}{s(d_i)}$$ Equation S4

r_i is the standardized residuals, d_i is the residual, which is calculated by doing the difference of the observed response y_i and the computed response $\hat{y}_i$ and s is the estimated sample standard variation.

V. References

1 K. Ingar Draget, K. Østgaard and O. Smidsrød, *Carbohydrate Polymers*, 1990, **14**, 159-178.

2 M. C. Daniel and D. Astruc, *Chem Rev*, 2004, **104**, 293-346.

3 N. Raval, R. Maheshwari, D. Kalyane, S. R. Youngren-Ortiz, M. B. Chougule and R. K. Tekade, in *Basic Fundamentals of Drug Delivery*, ed. R. K. Tekade, Academic Press, 2019, DOI: https://doi.org/10.1016/B978-0-12-817909-3.00010-8, pp. 369-400.
